# Exome resequencing and GWAS for growth, ecophysiology, and chemical and metabolomic composition of wood of *Populus trichocarpa*

**DOI:** 10.1186/s12864-019-6160-9

**Published:** 2019-11-20

**Authors:** Fernando P. Guerra, Haktan Suren, Jason Holliday, James H. Richards, Oliver Fiehn, Randi Famula, Brian J. Stanton, Richard Shuren, Robert Sykes, Mark F. Davis, David B. Neale

**Affiliations:** 1 Department of Plant Sciences, University of California at Davis, 262C Robbins Hall, Mail Stop 4, Davis, CA 95616 USA; 2 Instituto de Ciencias Biológicas, Universidad de Talca, Talca, P.O. Box 747, 3460000 Chile; 3 Department of Forest Resources and Environmental Conservation, Virginia Polytechnic Institute and State University, Blacksburg, VA 24061 USA; 4 Department of Land, Air and Water Resources, University of California, Davis, CA 95616 USA; 5 Department of Molecular and Cellular Biology & Genome Center, University of California, Davis, CA 95616 USA; 6 Biological Research Group, GreenWood Resources, Portland, OR 97201 USA; 7 National Renewable Energy Laboratory, Golden, CO 80401 USA; 8 Bioenergy Research Center, University of California at Davis, Davis, CA 95616 USA

**Keywords:** *Populus*, GWAS, Sequence capture, Growth, Stable isotopes, Lignin, Cellulose, Wood metabolome

## Abstract

**Background:**

*Populus trichocarpa* is an important forest tree species for the generation of lignocellulosic ethanol. Understanding the genomic basis of biomass production and chemical composition of wood is fundamental in supporting genetic improvement programs. Considerable variation has been observed in this species for complex traits related to growth, phenology, ecophysiology and wood chemistry. Those traits are influenced by both polygenic control and environmental effects, and their genome architecture and regulation are only partially understood. Genome wide association studies (GWAS) represent an approach to advance that aim using thousands of single nucleotide polymorphisms (SNPs). Genotyping using exome capture methodologies represent an efficient approach to identify specific functional regions of genomes underlying phenotypic variation.

**Results:**

We identified 813 K SNPs, which were utilized for genotyping 461 *P. trichocarpa* clones, representing 101 provenances collected from Oregon and Washington, and established in California. A GWAS performed on 20 traits, considering single SNP-marker tests identified a variable number of significant SNPs (*p*-value < 6.1479E-8) in association with diameter, height, leaf carbon and nitrogen contents, and δ^15^N. The number of significant SNPs ranged from 2 to 220 per trait. Additionally, multiple-marker analyses by sliding-windows tests detected between 6 and 192 significant windows for the analyzed traits. The significant SNPs resided within genes that encode proteins belonging to different functional classes as such protein synthesis, energy/metabolism and DNA/RNA metabolism, among others.

**Conclusions:**

SNP-markers within genes associated with traits of importance for biomass production were detected. They contribute to characterize the genomic architecture of *P. trichocarpa* biomass required to support the development and application of marker breeding technologies.

## Background

*Populus* species and their hybrids are suitable feedstocks for second-generation biofuel production due to their rapid growth rates and favorable cell wall chemistry [1, 2]. In particular, the model species *Populus trichocarpa* Torr. & A. Gray (black cottonwood), native to western North America, has been used in breeding for generating commercial cultivars [3]. Biomass yield and chemical quality of *P. trichocarpa* cultivars, as well as their improvement, depend on multiple biological and environmental factors [4]. Considerable phenotypic and genetic variation has been observed in *P. trichocarpa* for complex traits related to growth, phenology, morphology, ecophysiology and wood chemistry [5–10]. These phenotypes include diameter and height [11, 12], bud set and flush [6, 13, 14], leaf morphology [15], water-use efficiency (WUE) [16, 17], secondary xylem composition [18] and wood metabolome [5]. This sort of traits has been also correlated with environmental variables such as latitude, daylength and temperature [5, 6, 14–16, 19].

Association analyses based on SNPs have been applied in recent years to identify polymorphisms controlling variation in complex traits of interest for biofuel production in *Populus* species [9, 15, 18–21]. Different approaches (candidate gene or GWAS) as well as genotyping platforms have been used, with single SNP-markers accounting for, in general, a low percentage of the phenotypic variation (1–8%) in studied traits. These results support the polygenic nature and complexity of inheritance patterns and justifies increasing efforts to elucidate the genomic basis controlling those phenotypes.

Among “next-generation” sequencing alternatives, genome complexity reduction by sequence capture, or targeted sequencing, represents an efficient approach to performing genome wide analysis [22]. This method restricts attention only to specific genome regions (both genic and intergenic) of interest for molecular breeding as well as investigations into the diversity, population structure and demographic history of unstructured natural populations among others [23]. This approach has advantage of being quick, simple, and requires relatively small amount of input DNA [24]. Furthermore, compared with alternatives such as whole genome sequencing, it is reduced in terms of non-pertinent repetitive sequences, allows multiplexing of more samples for a given sequencing space, identifies functional molecular markers, provides high coverage for identification of low frequency sequence variants, and can circumvent problems arising from the presence of paralogous genes derived from duplication or polyploidization events [24]. This is particularly important for *Populus* species, which have experienced a whole-genome duplication event [25]. It was demonstrated by the application of an exome capture approach for analyzing the genomic architecture of clinal variation in *P. trichocarpa* [26].

In the present study, we employ sequence capture for genotyping and performing a GWAS in a *P. trichocarpa* population of 461 clones from 101 provenances collected from the Pacific Northwest (Oregon and Washington) in the United States. In an previous study [5], representatives of these clones were established in a clonal trial in California and characterized, both by traditional field measurements and high-throughput phenotyping, in describing a suite of traits involved in biomass production and wood chemical composition. Now, we coupled these phenotypic measures with specific exome capture-based genotyping to identify SNPs underlying observed trait variation. The association population was generated with germplasm collected from the southern part of the *P. trichocarpa* range in North America, and it was established and evaluated (at age two) in a trial located significantly to the south than that range. That represents a particular environmental/experimental condition, useful to determine, for example, the effects of geographic relocation on the *P. trichocarpa* performance. Understanding genetic variation at a genome-wide scale is fundamental for developing genome-based breeding technologies suitable for supporting the development of genetically improved plantations for bioethanol production.

## Results and discussion

We used GWAS to identify DNA polymorphisms associated with biomass production and wood chemical composition in *P. trichocarpa*, which determine its potential as feedstock for lignocellulosic ethanol. This approach complements our previous phenotypic characterization of the same association population [5] by identifying SNPs underlying traits of growth, ecophysiology and wood quality, the primary traits targeted for the development of genetically improved clones suitable for dedicated biomass and bioenergy plantations. An approach based on sequence capture allowed us to detect genotype-phenotype associations across the *P. trichocarpa* gene exome.

The association population used in this study consisted of 461 clones (from 101 provenances), comprising part of the natural distribution range of *P. trichocarpa* in the Pacific Northwest of the United States. In a previous study [5], we observed significant phenotypic and genetic variation for growth, spring bud phenology, water use efficiency, C and N assimilation, as well as lignocellulosic components and metabolome of wood (Table 1). Similarly, clonal repeatability, represented in terms of individual heritability estimates, also varied among the traits. We hypothesized from this information that multiple polymorphic loci across the genome should be detected in association with phenotypes, and particularly, those with high heritability should reveal a large number of significant SNP-markers.

**Table 1:** Summary statistics for traits studied in the *Populus trichocarpa* association population. Columns “Mean”, “Std. Dev.”, “C.V” and “ ${\hat{H}}_c^2$ ” were extracted from Guerra et al. [5]. "R.A.", Relative abundance

Trait		Unit	Mean	Std. Dev.	C.V. (%)	*H*^*2*^_*c*_
Growth	Diameter (DBH)	mm	53.2	7.9	14.8	0.52
	Height (h)	dm	67.1	4.1	6.1	0.42
	Volume index (Vol)	m^3^	0.016	0.005	31.3	0.53
Phenology	Days to bud flush (DBF)	Julian days	87.4	7.8	8.9	0.9
Ecophysiology	Leaf C content	% DW	44.4	1.6	3.6	0.09
	Leaf N content	% DW	3.2	0.3	9.4	0.28
	Leaf C:N ratio (C:N)	kg C/kg N	14.2	1.3	9.2	0.33
	Leaf Δ	‰	19.2	0.7	3.6	0.26
	Leaf δ ^15^N	‰	2.5	0.4	16.0	0.25
	Specific leaf area (SLA)	m^2^/kg DW	12	1.5	12.5	0.27
	N content: SLA ratio (NArea)	g N/m^2^	2.8	0.4	14.3	0.28
Wood chem. Components	Wood 5-carbon sugars (C5)	%	36	2.2	6.1	0.07
	Wood 6-carbon sugars (C6)	%	42.3	3.3	7.8	0.08
	Wood lignin	%	22.7	1	4.4	0.15
	Wood syringil:guayacil ratio (S:G)	fold	1.9	0.1	5.3	0.58
Wood metabolites	Galactonic acid (GAc)	R.A.	0.6	0.4	62.8	0.22
	Galactinol (Gal)	R.A.	144.1	75.0	52.0	0.28
	Alpha tocopherol (Toc)	R.A.	69.3	31.1	44.9	0.16
	Adenosine (Ade)	R.A.	2.8	1.0	33.4	0.25
	4-Hydroxybenzoic acid (HbA)	R.A.	5.9	4.6	78.3	0.45

### Genotyping

**Fig. 1:**
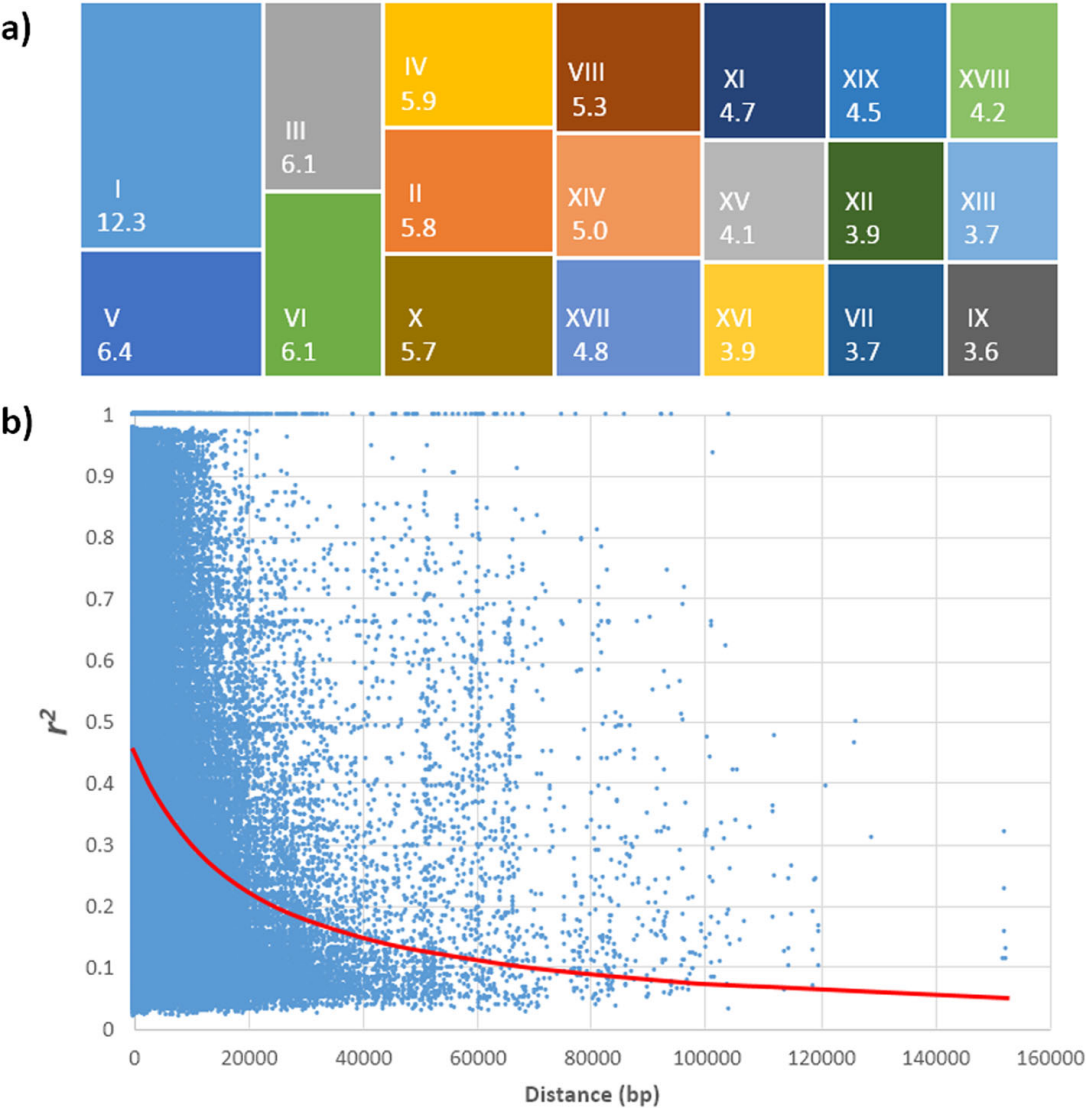
SNP genotyping and LD decay. **a** Relative contribution (in percentage) of each chromosome to the total (813,280) of analyzed SNP-markers. **b** Representative LD plot depicting the LD decay for Chromosome 12. The red line indicates the adjusted model for the significant correlations between SNP pairs

The processes of exome sequencing and genotyping identified 5.1 million SNPs across the *P. trichocarpa* genome in the association population, and after filtering, a set of 813,280 SNPs was used for association analyses (Table 2). The number of selected SNPs was proportional to chromosome size, ranging from 29,287 to 100,299 SNPs, for chromosomes 9 and 1, respectively (Table 2, Fig. 1a). Considering the full genome length, an average of one SNP every 482 bp (Table 2) was included in the analyses. Taking advantage of the full genome assembly, genotyping methodologies such as those based on sequence capture can target entire exons or genes across the genome, avoiding bias arising by a priori selection of candidate loci [23, 25]. In comparison to similar preceding studies that used SNP array platforms [6, 18, 19, 27], the number of SNPs in our analyses represent an increase in the power of applied genomic scanning. However, this amount is lower than the utilized by approaches based on whole-genome sequencing developed recently [7, 15].

**Table 2:** Summary of amount of analyzed SNP markers and intrachromosomal LD decay across the *Populus trichocarpa* genome. Linkage disequilibrium decay is referred to the physical distance (kbp) where LD = 0.2

Chr.	Size (Mbp)	Analyzed SNPs	Frequency (bp/SNP)	LD Decay (kbp)
1	50.5	100,299	503.4	29.99
2	25.3	47,563	531.1	27.49
3	21.8	49,962	436.7	27.19
4	24.3	47,671	509.1	22.36
5	25.9	52,236	495.6	23.35
6	27.9	49,374	565.3	27.21
7	15.6	30,295	515.3	18.85
8	19.5	43,099	451.6	21.99
9	12.9	29,287	442.1	21.63
10	22.6	46,758	482.9	24.21
11	18.5	38,563	479.8	51.63
12	15.8	31,964	493.1	25.13
13	16.3	30,493	535.2	28.07
14	18.9	40,482	467.4	29.65
15	15.3	33,418	457.2	18.85
16	14.5	32,006	452.9	26.22
17	16.1	39,114	411.1	33.83
18	17	34,049	498.1	33.39
19	15.9	36,647	435.0	19.26
Total	394.5	813,280	–	–
		Mean	482.3	26.86

### Intra-chromosomal linkage disequilibrium

The extent of linkage disequilibrium (LD) was analyzed across each chromosome. On average, the LD over physical distance decayed below *r*^*2*^ 0.2 at 26.9 kbp. A representative example, for Chromosome 12, is depicted in Fig. 1b. The complete set of chromosomes with its LD is included in Additional file 3: Figure S1. The decay varied depending on specific chromosomes, with the most rapid decay observed on chromosomes 7 and 15 (*r*^*2*^ 0.2 at 18.9 kbp) and the slowest decay on chromosome 11 (*r*^*2*^ 0.2 at 51.6 kbp). Genome-wide LD decay exhibited different extents among chromosomes (Table 2). LD decay to *r*^*2*^ < 0.2 was observed on average at 26.9 kbp. High variation of LD across the genome (among and within chromosomes) has been reported for this species [23]. The estimated extent of LD decay predicted in our study is higher than the observed by Wegrzyn et al. [18] (*r*^*2*^ 0.2 at ~ 0.5 kbp) and Wang et al .[28] (*r*^*2*^ 0.2 at ~ 8 kbp) for *P. trichocarpa.* Distinct methodologies, number of markers, population sizes, genetic origins and standard errors among the studies may account for the different findings. Compared with other tree species extent of LD estimated in this study is similar to species belonging to *Fraxinus* [29], *Prunus* [30] and *Eucalyptus* [31] genus.

### Single SNP-marker associations

**Fig. 2:**
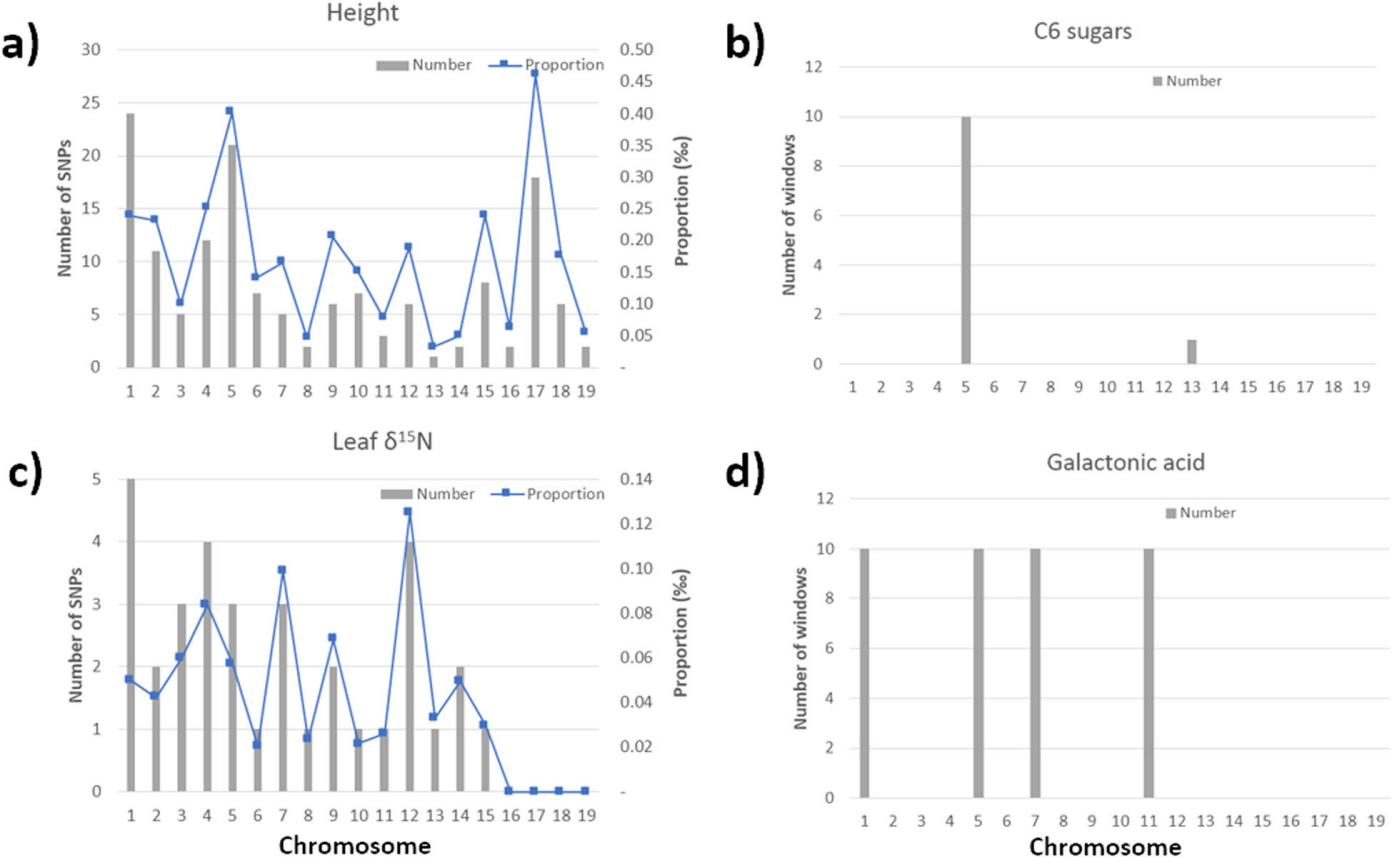
Number of significant single-SNPs (left) and sliding windows (right) associated with a selected set of traits for growth (**a**), stable isotopes parameters (**c**), chemical components of wood (**b**) and selected metabolites (**c**). Blue line at the left graphs indicates the proportion (‰) of significant SNP calculated on the total of analyzed SNP per chromosome. Significance thresholds considered a *p*-value < 6.1479E-8 for single-SNPs (**a** and **c**), and 1.04E-03 and 5.05E-04 for C6-sugars (**b**) and GAc (**d**) sliding windows, respectively. Detailed information is provided in Additional file 1: Tables S1 and S2

Significant associations (*p*-value < 6.1479E-8) were identified for DBH, h, leaf C and N content, and δ^15^N. Figure 2a and c depicts the number of associations detected per chromosome for a selected set of traits. A detailed list for each trait is provided in Additional file 1: Table S1. Similarly, Manhattan plots for each phenotype are included in Additional file 4: Figure S2.

In general, and consistently with chromosome length, the highest numbers of significant associations were observed for chromosomes 1 and 5. The lowest number of associations was observed for chromosome 16. The proportion of significant SNPs of the total analyzed, ranged from 0.02 ‰ to 0.50 ‰ for leaf C content on chromosome 10, along with δ^15^N on chromosomes 6 and 10, and leaf N content on chromosome 5 (Additional file 1: Table S1b), respectively. In the case of growth traits, 2 and 148 associations were detected for DBH and h, respectively. Within the ecophysiological traits, the number of significant associations ranged from 12 to 220 for C content and leaf N-content, respectively. For traits related to the chemical composition of wood, associated SNP-markers were over the significance cutoff (*p*-value < 6.1479E-8). Similarly, in the case of wood metabolites, considering a selected subset of those with the top five highest heritability estimates, no significant associations meeting the adjusted *p*-value were identified for Adenosine (Ade), Hydroxybenzoic Acid (HbA), Galactinol (Gal), Galactonic Acid (GAc) and Alpha tocopherol (Toc). The proportion of phenotypic variation accounted for the cumulative effect of significantly associated SNPs was 0.2, 1.1, 0.1, 0.7 and 0.7% for DBH, h, leaf C content, leaf N content, and δ^15^N, respectively.

**Fig. 3:**
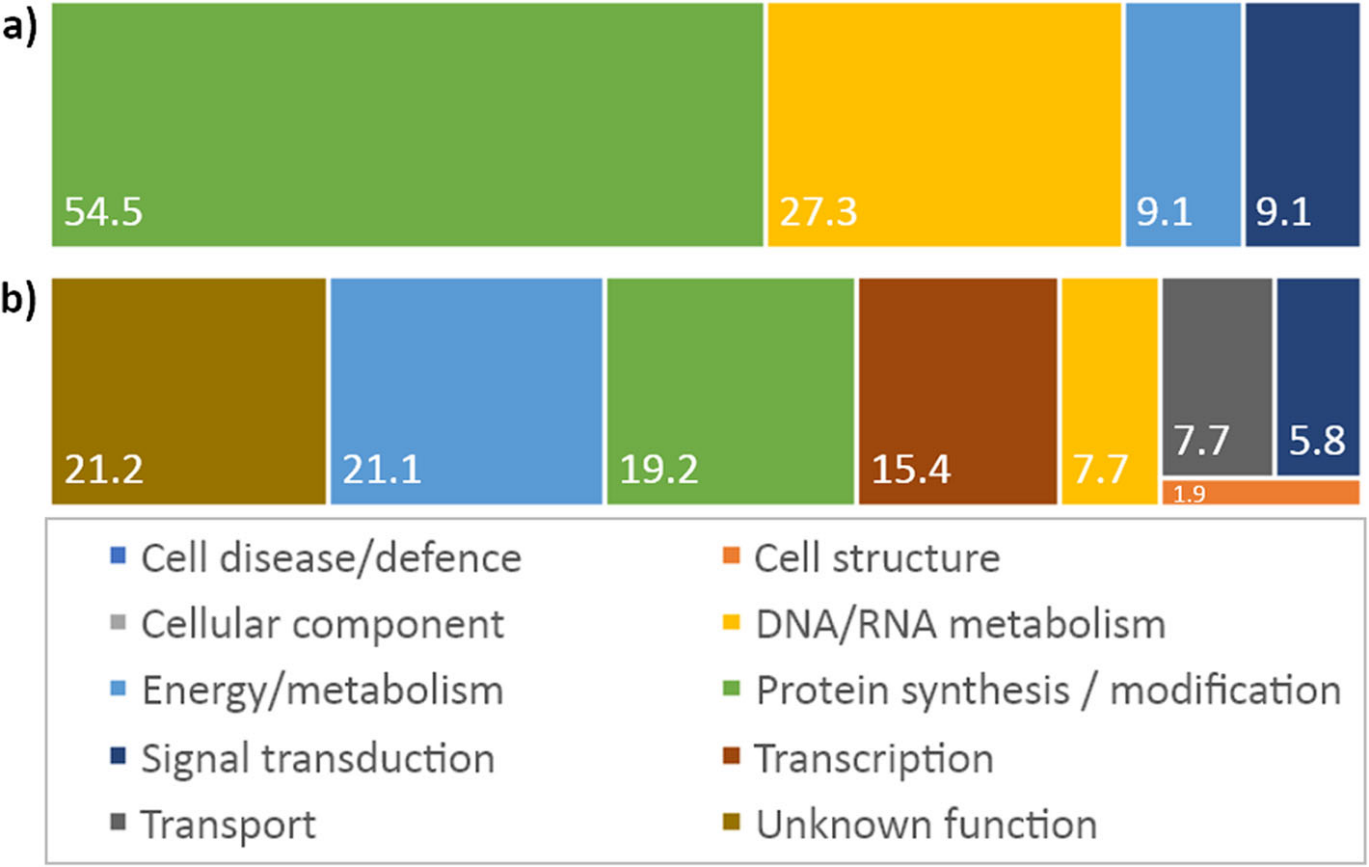
Main functional classes for the top three significant single-SNPs or sliding windows identified across all the analyzed phenotypes. **a** Single SNP-marker associations. **b** Sliding window analyses. Numbers represent percentages on total top three single-SNPs or sliding windows. Detailed information about specific SNP or windows is provided in Additional file 1: Tables S3 and S4

Significant single nucleotide polymorphisms associated with phenotype were identified mostly in exonic regions. SNPs are part of genes encoding proteins belonging to the functional classes: Protein Synthesis/Modification (54.5%), DNA/RNA Metabolism (27.3%), Energy/Metabolism (9.1%) and Signal transduction (9.1%) (Fig. 3a). A list with these SNPs and genes is given in Additional file 1: Table S3. An example for the Protein Synthesis/Modification category was a gene encoding a Periodic Tryptophan Protein 1 (Potri.007G019500), which was associated with height, and leaf N and δ^15^N. Among genes related with proteins involved in DNA/RNA Metabolism, one for a helicase senataxin (without gene model in Phytozome) was significant for height and leaf N. For genes in the Energy/Metabolism functional class, a representative was one (Potri.015G119700) encoding a Domain of unknown function (PGG), which was associated with DBH. For the Signal transduction class, the gene encoding a Rop Guanine Nucleotide Exchange Factor 1 (Potri.009G140100) was significant for height and leaf N.

Considering the applied significance threshold with Bonferroni correction (*p*-value < 6.1479E-8), GWAS performed on single-SNPs was successful in identifying polymorphisms associated with growth traits (DBH and h), leaf C and N-contents, as well as stable isotope parameters (δ^15^N) (Fig. 2, Additional file 1: Table S1). For traits related to spring bud phenology (DBF), wood chemical components (C5 and C6 sugars, lignin) and wood metabolites (GAc, Gal and HbA) significant associations at *p*-value< 0.0001 were detected, but they did not reach the adjusted threshold. The presence or lack of significant SNPs for these traits appears to be independent of heritability estimates for each. For some traits with moderate to high *H*^*2*^_*i*_ (e.g. S:G ratio or DBF), GWAS did not detect single-SNP associations. On the other hand, for traits with low to moderate *H*^*2*^_*i*_ (e.g. leaf C-content and δ^15^N) a relatively higher number of SNPs were identified. Similar situations were observed for phenology traits in previous studies with *P. trichocarpa* [19]. On average for all traits with significant associations ~ 1% of phenotypic variation was accounted for by the cumulative effect of significant SNPs. The influence of multiple SNPs associated with phenotypes is particularly interesting in the context of the development of models for genomic selection, where large numbers of markers are utilized to predict the genetic merit of individuals [32]. Differences among traits in terms of the number of significant SNP-markers suggest the differential effect of both the variable number of SNPs influencing each trait and the individual impact of some SNPs. In that sense, some individual SNPs could have a such low effect size that none reach statistical significance. Furthermore, the apparent lack of correspondence between estimates of *H*^*2*^_*i*_ and the phenotypic variance collectively accounted for by SNPs, could be explained by non-additive effects (e.g epistasis, GxE effect) or epigenetic factors acting on some traits. These types of effects are usually underestimated because MLM utilized for GWAS only suppose additive interactions [19]. Finally, another factor influencing the number of significant associated SNPs (and their effect on phenotypes) deals with the complexity of analyzing thousands of single markers across the genome. Stringent thresholds for controlling type I error are required for *p*-value adjustment in GWAS, given the correlated nature of markers along a chromosome [33]. For example, it has been suggested that the general applicability of the traditional false discovery ratio (FDR) [34] may suffer from several problems when applied to association analysis of a single trait [35]. In that sense, we utilized the Bonferroni correction to define the significance threshold. Thus, in spite of significant associations were detected at *p*-value < 0.00001 (and even lesser) in traits such as Vol, DBF, lignin or GAc, they did not reach the adjusted *p*-value threshold and were considered non-significant.

### Sliding window analyses

**Fig. 4:**
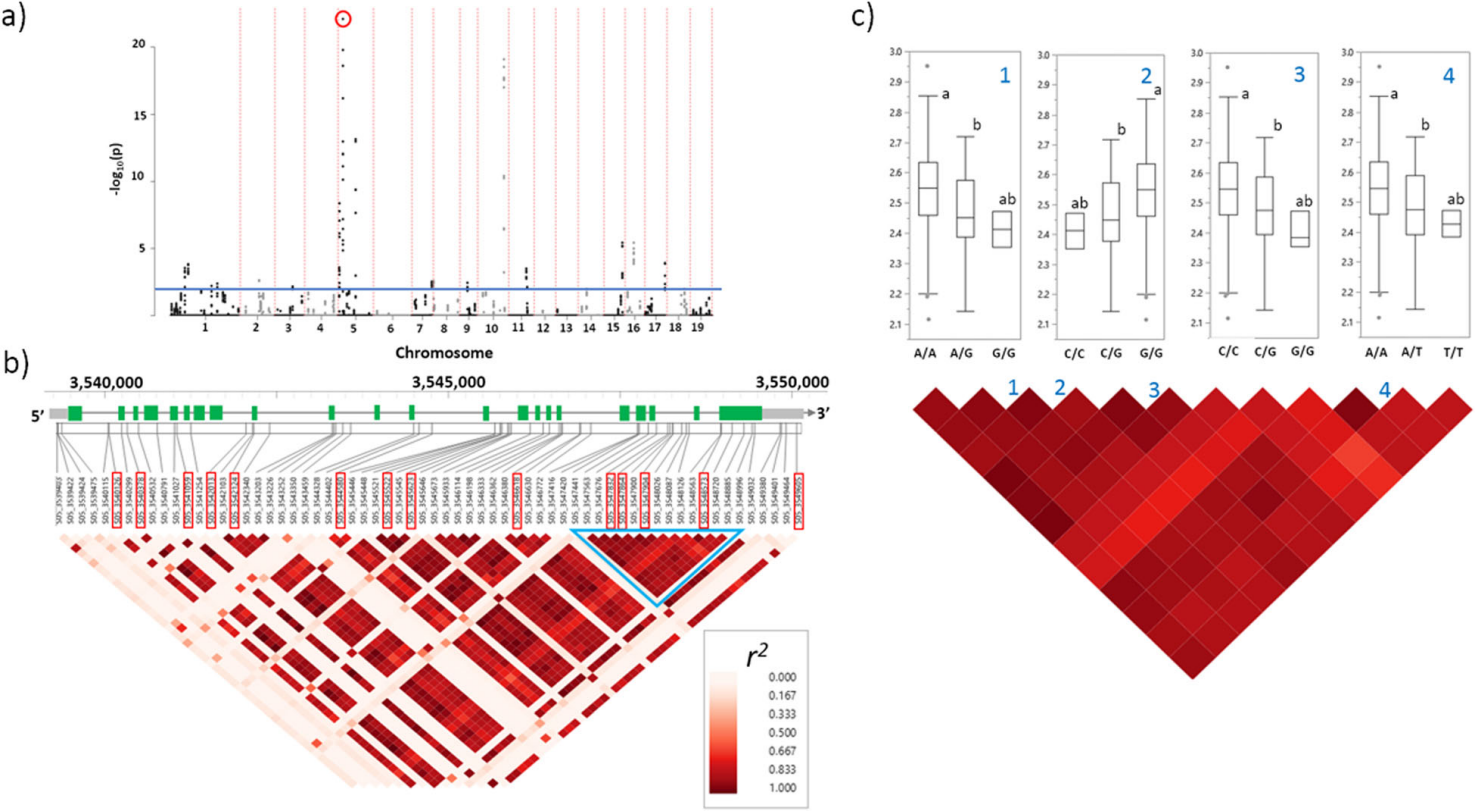
Detailed characterization of Similar to 5′-3′ Exoribonuclease (*XRN4*) gene (Potri.005G048900) associated with leaf δ^15^N. **a** Manhattan plot for leaf δ^15^N highlighting (red circle) the window containing significant SNPs for the gene. The horizontal blue line indicates a referential -log_10_ (*p*-value) of 2 (equivalent to *p*-value = 0.01). **b** LD heat map for the analyzed SNPs located at gene. Red bars at the top correspond to SNPs identified as significantly associated with δ^15^N by single-marker association tests. **c** Detailed view for the light blue triangle depicted in b). Numbers 1, 2, 3 and 4 are the markers S05_3547832, S05_3547864, S05_3547904 and S05_3548573, respectively. Boxplots shows the effects of genotypes on leaf δ^15^N. Different letters indicate significant differences among adjusted means (Tukey’s HSD test; *α* = 0.001)

The multiple-marker analysis by sliding-window allowed us to identify genomic regions containing different sets of SNPs jointly associated with each trait. Figure 4a depicts a representative Manhattan plot with the significant windows identified for leaf δ^15^N. Manhattan plots for other traits are included in Additional file 5: Figure S3. A variable number of windows per chromosome were detected among the phenotypes (Fig. 2b and d). The total number of significant windows ranged from 6 for HbA, to 192 for N content (Additional file 1: Table S2). For most traits, the main contributions were observed by chromosomes 8 and 1. However, for traits such as DBF, C:N, δ^15^N, and Toc, the most relevant chromosomes in terms of the number of significant windows included to 6, 4, 5 and 10, respectively. The multiple-SNP approach applied by sliding window analysis has been proposed as a robust alternative for identifying clustered significant patterns of SNPs, that are associated with complex traits, in a chromosomal context in humans and plants [36–39]. In our study, significant windows identified a series of SNP clusters which were coincident with coding regions of multiple genes (Additional file 1: Table S4). The graphical relationship between SNPs identified by single-marker associations and the detection by sliding window analysis is depicted in Fig. 4, where the highlighted window (Fig. 4a) contains 14 significant SNPs belonging to the *XRN4* gene (Fig. 4b). Additionally, information coming from both detection approaches allowed us to define genome zones with high LD, significantly associated with phenotypic variation, revealing the presence of phenotypically-relevant haplotypes (Fig. 4c). Although more evidence will be necessary, haplotype blocks defined by this way could be indicative of polymorphic regions with pleiotropic effects.

Considering the top three most significant windows across all chromosomes and traits, the most represented functional classes were Protein with Unknown Function (21.2%), Energy/Metabolism (21.1%), Protein Synthesis/Modification (19.2%), and Transcription (15.4%) (Fig. 3b). A list with the windows and genes included in these classes are given in Additional file 1: Table S4. Some of the detected genes encoding proteins with roles in Protein Synthesis/Modification were those expressing Similar to Threonyl-tRNA Synthetase (Potri.008G145600), Interleukin-1 Receptor-Associated Kinase 4 (Potri.008G145900) and Leucine Rich Repeat (LRR1) (Potri.005G015700) associated with wood C5-sugars, C6-sugars and height, respectively. An example of the genes dealing with proteins belonging to the Energy/Metabolism class were Exostosin Heparan Sulfate Glycosyltransferase-Related (Potri.010G197900) and Similar to Aldehyde Dehydrogenase 1 Precursor (Potri.012G078700), associated with δ^15^N and DBF, respectively. Among genes encoding enzymes involved in Transcription, Similar to Agamous-like MADS Box Protein AGL12 (Potri.019G076800) and WRKY Transcription Factor 10-related (Potri.013G086000), were associated with Gal and C5-sugars, respectively. Concerning the Protein Synthesis/Modification class, examples of identified genes are those encoding a Similar to Threonyl-tRNA synthetase and Leucine Rich Repeat (LRR_1)//Leucine rich repeat (LRR_8), associated with C5-sugars and height, respectively.

### Genes detected by single-SNP association and sliding windows approaches

We also verified the consistency between SNP-markers identified by single-SNP association and sliding window analyses. To this end, we considered as an example the most significant window (#154; *p*-value 4.70E-25) detected in chromosome 5 for leaf δ^15^N (Fig. 4a). The SNPs identified within the window were part of a gene (Potri.005G048900) encoding a Similar to 5′-3′ Exoribonuclease (XRN4) Gene, which is involved in disease resistance, response to ethylene, RNAi, and miRNA-mediated RNA decay [40]. The associated window comprised 64 SNPs (Fig. 4b). Fourteen of these markers, were also identified by the single-marker association, indicating the consistency between both approaches. Particularly, markers such as S05_3547832, S05_3547864, S05_3547904 and S05_3548573 were in high LD (*r*^*2*^ > 0.75) and produced significant variation at the level of leaf δ^15^N means (Fig. 4c). Alternatively, alleles for those SNPs represent intronic and non-synonymous polymorphisms, involving possible effects on transcript splicing, and protein structure and function.

Significant associations for traits underlying growth, nutrient metabolism and xylem formation, among others, define SNPs and genes, which might represent logical candidates for functional studies focused on confirming their role and impact on phenotype of *Populus* species. Considering their high significance and the simultaneous detection by the single-SNP association and/or sliding windows approaches, we centered part of our analysis on gene Similar to 5′-3′ Exoribonuclease (*XRN4*), which included SNPs and windows associated with leaf δ^15^N. The exoribonuclease function of this gene links it to transcription, RNA metabolism and RNA interference in eukaryotes [41]. In plants, it has been related to ethylene signaling [42] and response of plants to abiotic and biotic stresses [40, 43]. Mutation of members of the *XNR* gene family produced sensitivity to N starvation in *Saccharomyces cerevisiae* [44] and morphological alterations in *Arabidopsis thaliana* [45, 46]. Thus, association of *XRN4* with leaf δ^15^N, an indicator of N use efficiency [47], could be related to the N metabolism and mobilization at leaves, particularly during the last third of the growing season, when sampling was done. More studies will be required to detect possible effects of SNPs at *XRN4* on photosynthesis and biomass production.

### Chemical composition of wood

**Fig. 5:**
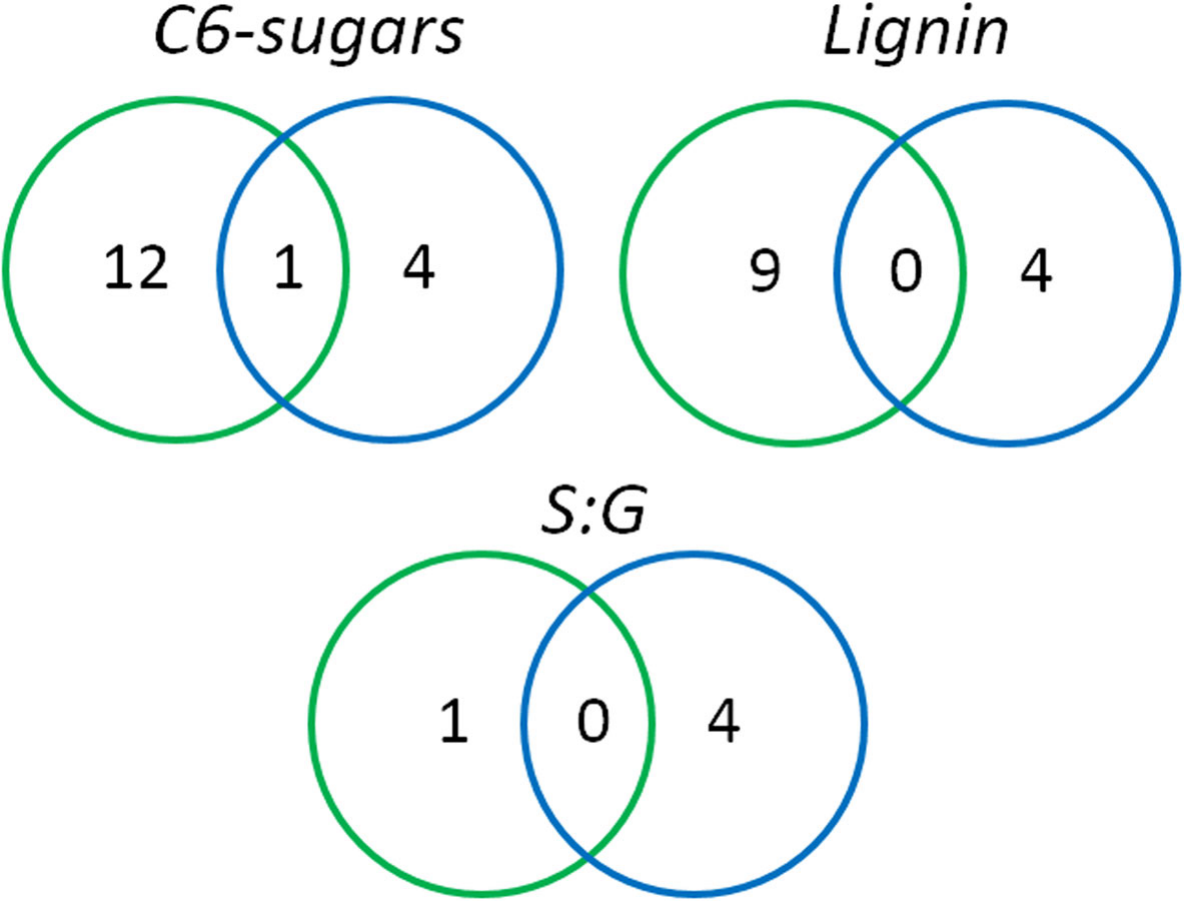
Venn diagrams for the comparison between the present study (blue circles) and the one carried out by Wegrzyn et al .[18] (green circles). Forty genes encoding enzymes involved in lignin and cellulose biosynthesis and cytoskeletal proteins were compared. Numbers in circles indicate the number of genes containing significant SNPs (*p* < 0.0001). A detailed list is given in Additional file 2: Table S5

Association analyses of the chemical composition of *P. trichocarpa* wood was previously performed in the same population by our group [18] using a candidate gene approach. We carried out a comparison between the results from both studies considering the 40 candidate genes utilized by Wegrzyn et al. [18], which encodes enzymes from the cellulose and lignin biosynthesis pathways and cytoskeletal proteins. Association results in the present study were significant only under a *p*-value < 0.0001 threshold. Results indicated both overlap and divergence between the two studies (Fig. 5 and Additional file 2: Table S5). SNPs within genes encoding cellulose synthase (*CesA1A*) were significantly associated with C6-sugars in both studies. For this trait, we also detected SNPs in *TUA5* gene, which were not identified by Wegrzyn et al. [18]. In the case of lignin content, members of the cellulose synthase gene family (*CesA2B* and *CesA1A*) were differentially detected (differentially) in both studies. We also identified SNPs belonging to 4- Serine Hydroxymethyl Transferase SHMT6. Finally, for the S:G ratio, our analyses detected SNPs in Laccase *LAC1A*, Phenylalanine Ammonia-Lyase *PAL5* genes, which were not identified by Wegrzyn et al. (2010). In spite of the genotypes and wood chemical characterization methods were mostly the same in both studies, distinct trial sites (the first in Westport, Oregon, and the second in Davis, California), sampling height or differential presence of juvenile wood, among other factors, might explain the differences in the findings reported previously [18] and those described in the present work.

## Conclusions

Forest trees are an important source for multiple wood and non-wood products. Genetic improvement aimed to develop such products, including lignocellulosic biofuels, depends on the variation underlying commercial traits. This variation is characterized by a complex genetic control and the influence of environmental factors. In this study, we identified a series of DNA polymorphisms controlling the phenotypic variation of growth, nitrogen use and wood composition of *P. trichocarpa* clones at different levels. Our results thus provide a starting point to define candidate genes suitable for functional characterizations addressed to confirm their biological role. At the same time, the genome-wide scale applied for the association analyses revealed a large number of SNPs which could be utilized to develop genomic selection schemes. Further efforts to define the utility of SNP polymorphisms in generating genomic breeding values will illuminate the path to breeding programs that incorporate molecular markers for bioethanol production. The upshot will be the estimation of parental hybridization values and an earlier, more precise genotypic selection from segregating F1 hybrid populations, that together will increase the overall magnitude of the realized genetic gain per unit of time.

## Methods

### Association population, growth conditions and phenotypic characterization

The association population was comprised of a set of 461 *P. trichocarpa* clones. These represented 101 provenances, within 14 river systems located west of the Cascade Mountains in Oregon and Washington between 48°54′ N latitude (Nooksack River, Whatcom County, Washington) and 43°47′ N latitude (Middle Fork, Willamette River, Lane County, Oregon) collected by GreenWood Resources [18]. A clonal trial was established at the University of California, Davis, California (38°32′42″ N, 121°47′42″ W) for phenotypic measurements and sample collection. The experiment was located on a silt loam soil (Entisol, Yolo series). Plants were produced from rooted containerized cuttings and established in April 2009, in an array of 1.83 × 1.83 m, following a randomized block design, with three blocks and one ramet per clone per block. Blocks were considered to control a north–south variation in the trial, associated with non-homogenous soil conditions. From 2009 to 2011, plants were irrigated once a week from June to September each year. No fertilizer was applied during the study.

The clonal trial was already characterized by Guerra et al. [5], in terms of traits dealing with growth, spring bud phenology, ecophysiology, and the chemical composition and metabolome of wood. Table 1 summarizes main statistics and clonal repeatability (heritability) estimates for those traits. A brief description about their measurements is indicated in the following subsections. Individual broad-sense heritability (*H*^*2*^_*i*_) was estimated using formula (1) [5]:

1 $$ {\hat{H}}_i^2=\frac{\ {\hat{\sigma}}_g^2+{\hat{\sigma}}_c^2+{\hat{\sigma}}_{e{e}^{\prime }}\ }{\ {\hat{\sigma}}_g^2+{\hat{\sigma}}_c^2+{\hat{\sigma}}_{g\mathrm{x}b}^2+{\hat{\sigma}}_e^2}, $$

where *σ*^*2*^_*g*_, *σ*^*2*^_*c*_, *σ*^*2*^_*f*x*b*_ and *σ*^*2*^_*e*_ represent the variance due the genetic cluster, clone within cluster, cluster x block and residual, respectively, and *σ*^*2*^_*ee’*_ is the covariance between residuals of the same clone in two blocks.

Data used in the phenotypic characterization is available at the TreeGenes platform (http://dendrome.ucdavis.edu/treegenes/) under the accession number TGDR050.

### Diameter, height, volume and spring-bud phenology measurements

Three growth parameters (diameter at breast height [DBH], total height [h] and volume index [Vol]), at age two, were measured in October 2010, as described by Guerra et al. [5]. In particular, Vol was estimated as Vol = π (DBH/ 2)^2^× h [13]. Additionally, days to bud flush (DBF), were recorded every 3 days from March to April 2011, as indicated previously [5].

### Chemical composition and metabolome of wood

Wood cores collected from tree stems (0.3 m above ground level), at age three, in September 2011, were utilized for analyzing the chemical composition and metabolome of wood [5]. The content of 5 and 6-carbon sugars and lignin, as well as the syringyl:guaiacyl monolignol ratio (S:G) were determined from wood cores by high-throughput pyrolysis molecular beam mass spectrometry (pyMBMS), at the National Renewable Energy Laboratory (Golden, CO, USA). Simultaneously, wood metabolites were quantified from another set of wood cores by gas chromatography coupled with time-of-flight mass spectrometry (GC-TOF-MS), at the West Coast Metabolomics Center, at UCDavis. Methodological details have been described elsewhere [5]. For association analysis, five metabolites were selected. These corresponded to those with the highest estimates of heritabilities according to Guerra et al. [5]: 4-hydroxybenzoic acid (HbA; ${\hat{H}}_i^2=0.45$), galactinol (Gal; ${\hat{H}}_i^2=0.28$), adenosine (Ade; ${\hat{H}}_i^2=0.25$), galactonic acid (GAc; ${\hat{H}}_i^2=0.22$), and alpha tocopherol (Toc; ${\hat{H}}_i^2=0.16$). These estimates involved a significant genetic variation across the analyzed genotypes, indicating their importance on wood composition variation. They were selected to maximize the power for detecting significant associations.

### Ecophysiology traits

Morphological and ecophysiological characteristics are determinants of biomass productivity [4]. For that reason, independent leaf samples were obtained from the top of each tree and used for stable isotope analyses and leaf area estimation, respectively. This sort of measurements was carried considering their relationship with tree physiology and they are easy to collect (compared to physiological traits). Sampled leaves collected in August 2011 were processed to determine carbon (C) and nitrogen (N) concentrations and C and N stable isotope compositions by continuous flow isotope ratio mass spectrometry, at the UCDavis Stable Isotope Facility, as described elsewhere [5]. In this stage, the 461 clones were sampled. Additionally, a complimentary second subset of leaves, collected in August 2012, was utilized for measuring leaf area and dry biomass to estimate the specific leaf area (SLA) and N content per SLA ratio (NArea), according to Easlon et al. [48]. In this case, the subset of leaves was obtained from 177 clones, including three replicates per clone (one per block). These clones were chosen including those with extreme geographical origins and minimal and maximal volume indices. Thus, from these measurements, a set of variables were generated, including: C and N content, C:N ratio, carbon isotope discrimination (Δ), δ^15^N, SLA, and NArea.

### DNA isolation, sequence capture and sequencing

Additional young leaves were collected for DNA isolation. Circle punches were obtained and deposited in plastic vials, along with silica gel packets, until desiccation. This sort of leaf samples is compatible with automated grinding and pipetting systems. DNA isolation was performed using the Qiagen DNeasyPlant Mini Kit (Qiagen, Inc., Valencia, California, USA), according manufacturer’s instructions. Quality was assessed with spectrophotometer (NanoDrop, Thermo Scientific). Acceptable extractions had a 260/280 ratio of 1.7–2.0 with a minimum concentration of 20 ng/μl.

Phytozome version 7.0 annotation and assembly files for *P. trichocarpa* (corresponding to assembly version 2.0 of the black cottonwood genome) were used to design oligonucleotide baits, complementary to short genomic regions that targeted exons, promoter, and intergenic control regions, as described by Zhou et al. [23]. A total of 230,720 baits of 120 bp were designed using SureSelect eArray software (Agilent Technologies, Santa Clara, California, USA). The baits targeted more than 39,000 of the 40,668 annotated protein-coding transcripts of the *P. trichocarpa* genome. As the cumulative length of the predicted exons exceeded the available baits, following bait design in eArray, we looped through the gene list, selecting one bait for each gene at each pass, until the maximum number of baits (i.e. 230,720) was reached. In addition to exons, baits were included in the design for genes with an annotated 5′-UTR targeting the 240 bp upstream, as well as 1000 baits targeting intergenic regions to be used as selectively neutral control regions. These control regions were selected at random from non-repetitive intergenic intervals at least 1000 bp from any gene model. This strategy of bait design has demonstrated previously that capture efficiency has not been significantly impacted by the presence of paralogous genes [25]. After design, a custom biotinylated RNA bait library was synthesized. Library preparation and target enrichment were performed following the Agilent SureSelect^XT^ protocol (Version B). Briefly, 3.0 μg of poplar genomic DNA was sheared on a ultrasonicator (Covaris S220) at the Virginia Bioinformatics Institute (VBI), followed by end repair, 3′-end adenylation, adaptor ligation, and amplification. Agencourt AMPure XP beads were used to purify the libraries following each step, and library quality was assessed using an Agilent Bioanalyzer 2100 instrument, with Agilent DNA 1000 chips. Samples were randomly assigned one of 96 available index sequences and subsequently randomly assigned to groups of 16, each of which corresponding to a HiSeq sequencing lane. The prepared libraries were hybridized to the RNA baits in solution at 65 °C in an Eppendorf Mastercycler PCR machine (Eppendorf, Hamburg, Germany), and subsequently purified on magnetic beads. The multiplexed libraries were sequenced using an Illumina HiSeq 2500 System in a 2 × 100 paired-end format at VBI. Sequences of the prepared libraries are available at the Sequence Read Archive (http://www.ncbi.nlm.nih.gov/Traces/sra/) under the accession number SRA058855.

### Data analysis and SNP calling

Short reads from poplar samples were pre-filtered using a collection of scripts in Biopieces (https://github.com/maasha/biopieces; version 2.0). First, interleaved pair-ended sequences were filtered based on the Illumina filter flag, and subsequently trimmed of adapters and bases with quality < 35. Following trimming, very short reads were eliminated (length < 35) to prevent ambiguous alignments. Lastly, reads having poor local quality scores (score < 25, window size = 5) were removed from the analysis. The short reads were aligned to the *Populus trichocarpa* (version 3.0) reference genome with the Burrows-Wheeler Aligner *mem* algorithm [49]. Resulting alignment files (in Sequence Alignment/Map, SAM, format) were converted via SAMtools v3.1 [50] to their binary versions (Binary Alignment/Map, BAM) for variant calling. Prior to SNP calling, duplicate reads were identified and removed using Picard software (version 2.6; https://broadinstitute.github.io/picard/command-line-overview.html) and the Genome Analysis Toolkit v3.x (GATK; https://software.broadinstitute.org/gatk/), with MarkDuplicates and DuplicateReadFilter functions, respectively [51]. Indels were realigned using the GATK IndelRealigner function. The HaplotypeCaller algorithm of GATK was then used to call SNPs (options: *min_base_quality_score > 9*, and *standard_min_confidence_threshold_for_calling > 29*) [52]. Variant calling was performed on individual chromosomes and scaffolds to reduce the run time. After merging all variant calling format (VCF) files, the VariantsToTable tool of GATK was used to produce SNP tables for downstream analysis. SNPs with a minor allele frequency < 5% and departure from Hardy-Weinberg equilibrium were excluded from the analyses. Similarly, SNPs that were missing data across more than 5% individuals (clones) were also removed from following stages. After these filtering steps, from 5.1 million identified SNPs, a final set comprised of 813,280 SNPs was utilized for GWAS.

### GWAS analyses

The distributions of the different traits were checked for departures from normality. Logarithmic transformations were applied to normalize the variables Vol, DBF, C and N concentration, C/N ratio, SLA, NArea, lignin, C5-sugars and C6-sugars. Outlier observations were excluded from tests. Clonal means were adjusted by Best Linear Unbiased Predictor, using Proc MIXED in the software SAS v9.2 (SAS Institute, Cary, NC, USA), in order to correct a significant block effect observed across the trial area. Prediction model included the factors clone and block, considered as random and fixed effects, respectively. The GWAS was based on Mixed Linear Models (MLM), implemented in the software GCTA v1.25 [53] (http://cnsgenomics.com/software/gcta/index.html). SNP data were first converted to PLINK format using TASSEL v3.0 [54] (http://www.maizegenetics.net/tassel), and then converted to binary PLINK (bed file) using PLINK v1.9 command line tool [55]. The genetic relatedness (kinship) was determined by the genetic relationship matrix (GRM) option at the GCTA’s GRM module, based on identical-by-descent estimates [56]. The population structure matrix (Q) was estimated by a classical multidimensional scaling [57], also called Principal Coordinates Analysis, using the *cmdscale* function in the package *Stats* in R [58]. With the marker (M), kinship (K), population structure (Q) and predictor variables, we ran an “M + K + Q” linear mixed model to identify significant associations with traits. Marker and Q were assumed fixed effects, whereas K represented a random effect. The goodness of fit including (or not) Q was assessed for each trait by the Bayesian information criterion (BIC), utilizing the package *Stats* in R [58]. Associations were adjusted for multiple testing, using the Bonferroni correction, in the package *Stats* in R [58]. The threshold for *p*-values was 6.1479E-8 at significant level of 5% after Bonferroni correction (0.05/813,280). We additionally used the Random Forest method to estimate the percentage variance explained by the top SNPs for each trait (SNP contribution), by using the *randomForest* package in R [59]. The SNP contribution standard deviation (SCSD) was also calculated by the same package. We set the number of trees (*ntree*) grown as 2000 and averaged 50 repeats per trait in our estimates.

Multiple-SNP testing for each trait was carried out using an overlapping sliding-window analysis on association results, based on the number of the significant associations (*p*-value < 0.00001) for 10 k window size with 1 k slide, using a custom script. Empirical *p*-values for each slide were estimated assuming the significant slides follow a Poisson distribution [36]. To this end, Poisson tests were carried out by comparing the rate of total significant SNPs and the total number of SNPs with the similar rate for the tested window using the probability formula below.

$$ P=\frac{{\mathrm{\gimel}}^k{e}^{-\mathrm{\gimel}}}{k!}, $$

Where lambda (ℷ) is the average number of significant SNPs, *e* is the constant Euler’s number and k is the average number of significant SNPs for the window being tested. Empirical *p*-values were recorded for each window per trait and then were adjusted by Bonferroni (0.05/number of tested windows) and reported for each trait. Finally, *p*-values were visualized using a Manhattan plot, highlighting only the significant slides.

### Linkage disequilibrium

Linkage disequilibrium (LD) was estimated among pairwise combinations of SNPs per chromosome. It was expressed in terms of the squared correlation of allele frequencies *r*^*2*^. The *r*^*2*^ value between pairs of SNP markers, within each chromosome, was estimated using TASSEL 5.2 [54], utilizing the option of sliding window (120 SNPs per window). To assess the extent of LD, the decay of LD within physical distance (base pairs) between SNPs, within each chromosome, was evaluated by nonlinear regression analysis of *r*^*2*^ values [60]. Analysis was performed applying the NLIN procedure in SAS 9.4.

### Gene models, annotations and expression data

Gene models and gene ontologies were obtained from the Phytozome platform version 12 (*Populus trichocarpa* v 3.0) and Quick GO site (http://www.ebi.ac.uk/QuickGO-Beta/), respectively.

## Supplementary information

**Additional file 1: Table S1.** a Number of significant single SNP-markers. b. Proportion of significant SNPs on total analyzed SNPs. **Table S2.** Number of significant sliding windows. **Table S3.** Top three significantly associated single SNPs. **Table S4.** Top three significantly associated SNP-windows.

**Additional file 2: Table S5.** SNPs belonging to genes encoding enzymes for lignin and cellulose biosynthesis pathways.

**Additional file 3: Figure S1.** Linkage disequilibrium decay per chromosome.

**Additional file 4: Figure S2.** Manhattan plots for assessed traits.

**Additional file 5: Figure S3.** Manhattan plots for sliding window analysis tests.

## Data Availability

Sequences of the prepared libraries for exome capture are available at the Sequence Read Archive (http://www.ncbi.nlm.nih.gov/Traces/sra/) under the accession number SRA058855. Data used in the phenotypic characterization is available at the TreeGenes platform (http://dendrome.ucdavis.edu/treegenes/) under the accession number TGDR050.

## Abbreviations

Ade: Adenosine
C:N: Leaf C:N ratio
C5: Wood 5-carbon sugars
C6: Wood 6-carbon sugars
DBF: Days to bud flush
DBH: Diameter
GAc: Galactonic acid
Gal: Galactinol
GWAS: Genome wide association study
h: Height
H^2^_i_: Individual broad-sense heritability
HbA: 4-Hydroxybenzoic acid
LD: Linkage disequilibrium
Narea: N content: SLA ratio
S:G: Wood syringil:guayacil ratio
SLA: Specific leaf area
SNP: Single Nucleotide Polymorphism
Toc: Alpha tocopherol
Vol: Volume index
Δ: Carbon isotope discrimination
