# Supplementary material for: Exome resequencing and GWAS for growth, ecophysiology, and chemical and metabolomic composition of wood of Populus trichocarpa
Source: BMC Genomics. 2019 Nov 20;20:875. doi: 10.1186/s12864-019-6160-9 (PMC6864938; doi:10.1186/s12864-019-6160-9)
Supplement: Supplementary file 2 — Additional file 2: Table S5. SNPs belonging to genes encoding enzymes for lignin and cellulose biosynthesis pathways. [file 12864_2019_6160_MOESM2_ESM.pdf]

Table S5. Number of SNPs belonging to genes encoding enzymes for lignin and cellulose biosynthesis pathways (referred in Wegrzyn et al. 2010), identified in the present study in association with wood chemical components. All associations were significant at p<0.0001.

Gene product	Gene	Gene model	Chr.	C6	Lignin	S:G
4-Coumarate:CoA ligase	*4CL1*	Potri.006G169600	6			
	4CL3	Potri.001G036900	1			
	4CL5	Potri.003G188500	3			
Coumarate 3-hydroxylase	*C3H3*	Potri.006G033300	6			
Cinnamate 4-hydroxylase	*C4H1*	Potri.019G130700	19			
	C4H2	Potri.013G157900	13			
Cinnamyl alcohol dehydrogenase	*CAD*	Potri.009G095800	9			
Cinnamoyl-CoA reductase	*CCR*	Potri.003G181400	3			
Cellulose synthase	*CesA1A*	Potri.011G069600	11	1		
	CesA1B	Potri.004G059600	4			
	CesA2A	Potri.018G103900	18			
	CesA2B	Potri.006G181900	6			
	CesA3A	Potri.002G257900	2			
Hydroxcinnamoyl-CoA transferase	*HCT1*	Potri.003G183900	3			
	HCT6	Potri.001G042900	1			
Cellulase	*KOR1*	Potri.001G078900	1			
Laccase	*LAC1A*	Potri.016G112000	16			1
	LAC2	Potri.008G064000	8			
	LAC90A	Potri.008G073700	8			
Phenylalanine ammonia-lyase	*PAL2*	Potri.008G038200	8			
	PAL4	Potri.010G224100	10			
	PAL5	Potri.010G224200	10			1
S-Adenosylmethionine synthetase	*SAM1*	Potri.008G099300	8			
Serine hydroxymethyl transferase	*SHMT1*	Potri.001G320400	1			
	SHMT3	Potri.002G109200	2			
	SHMT6	Potri.017G059300	17		1	
Sucrose synthase	*SUSY1*	Potri.018G063500	18			
α-Tubulin	*TUA1*	Potri.002G111900	2			
	TUA5	Potri.009G085100	9	1		
β-Tubulin	*TUB15*	Potri.001G272800	1			
	TUB16	Potri.009G067100	9			
	TUB9	Potri.001G104600	1			
Caffeoyl CoA O-methyltransferase	*CoAOMT1*	Potri.009G099800	9			
	CoAOMT2	Potri.001G304800	1			
Caffeate O-methyltransferase	*COMT1*	Potri.015G003100	15			
	COMT2	Potri.012G006400	12			

Ferulate 5-hydroxylase	*F5H1*	Potri.005G117500	5			
	F5H2	Potri.007G016400	7			
Glycine decarboxylase complex, H	*gdcH1*	Potri.012G123700	12			
Glycine decarboxylase complex, T	*gdcT2*	Potri.004G009600	4			
			**Total**	**2**	**1**	**2**
