# Supplementary figures and images for: Exome resequencing and GWAS for growth, ecophysiology, and chemical and metabolomic composition of wood of Populus trichocarpa
Source: BMC Genomics. 2019 Nov 20;20:875. doi: 10.1186/s12864-019-6160-9 (PMC6864938; doi:10.1186/s12864-019-6160-9)

**Figure S1.** Linkage disequilibrium decay by chromosome.

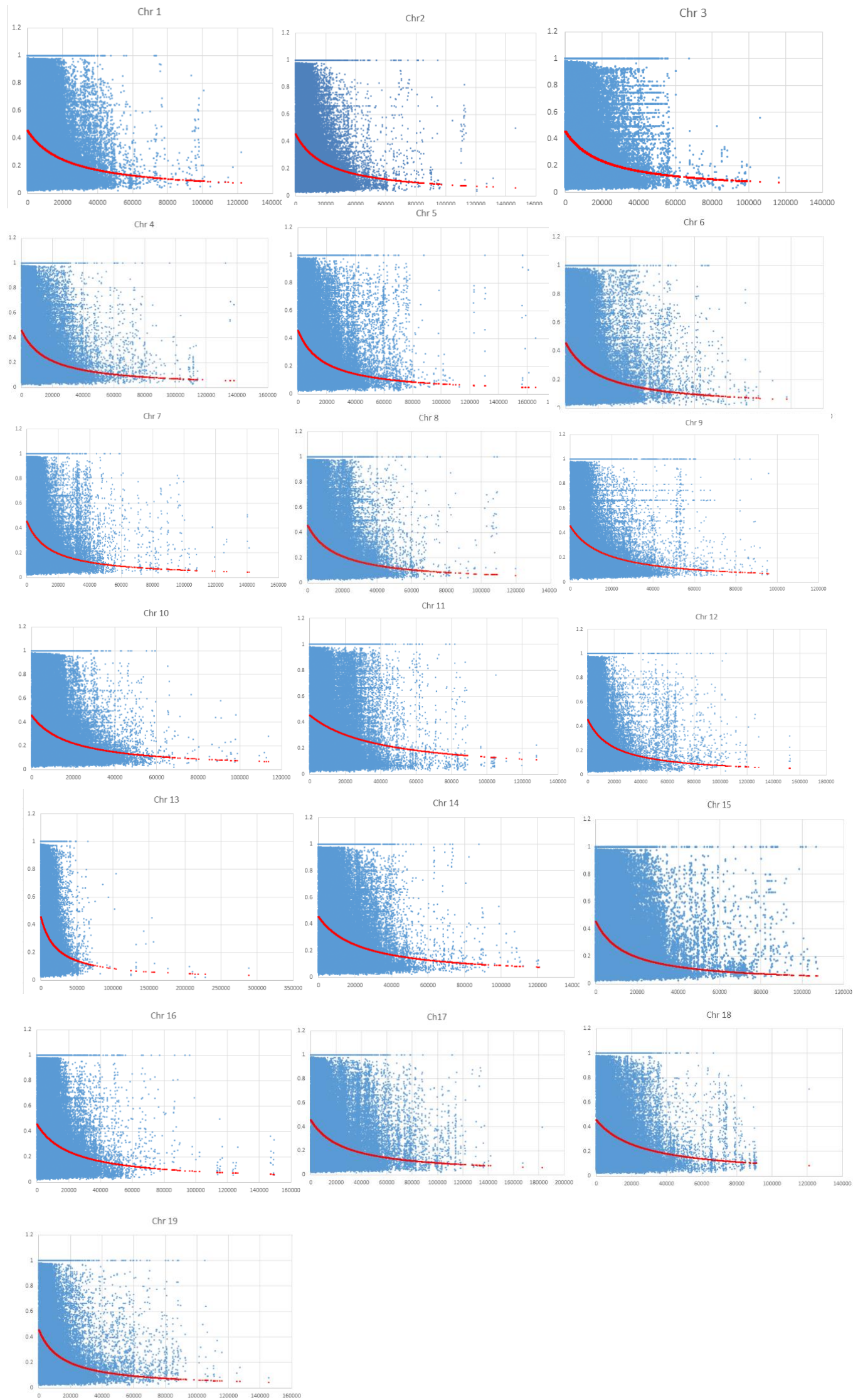

Supplement: Supplementary file 3 — Additional file 3: Figure S1. Linkage disequilibrium decay per chromosome. [file 12864_2019_6160_MOESM3_ESM.pdf]

**Figure S2.** Manhattan plots for single-marker tests

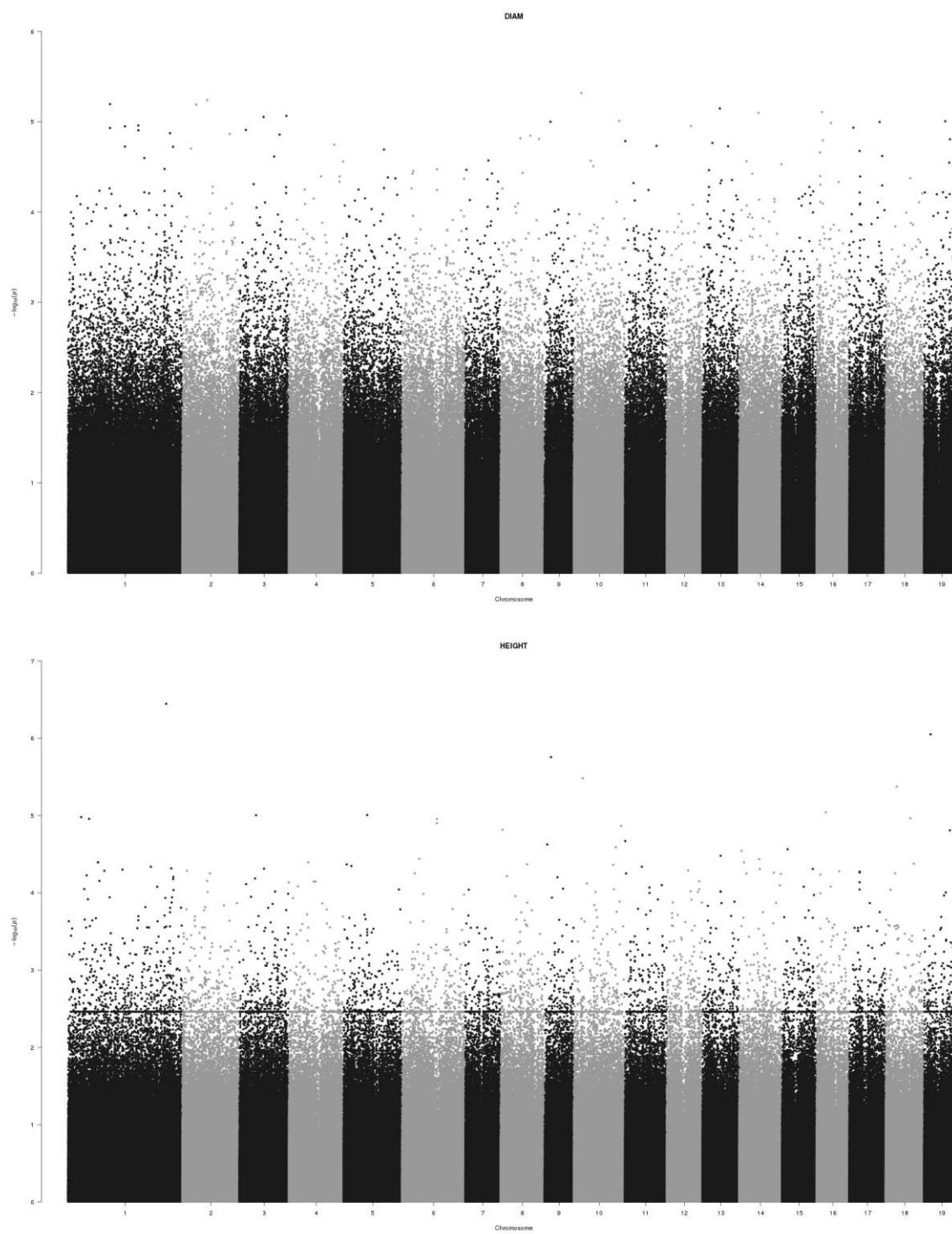

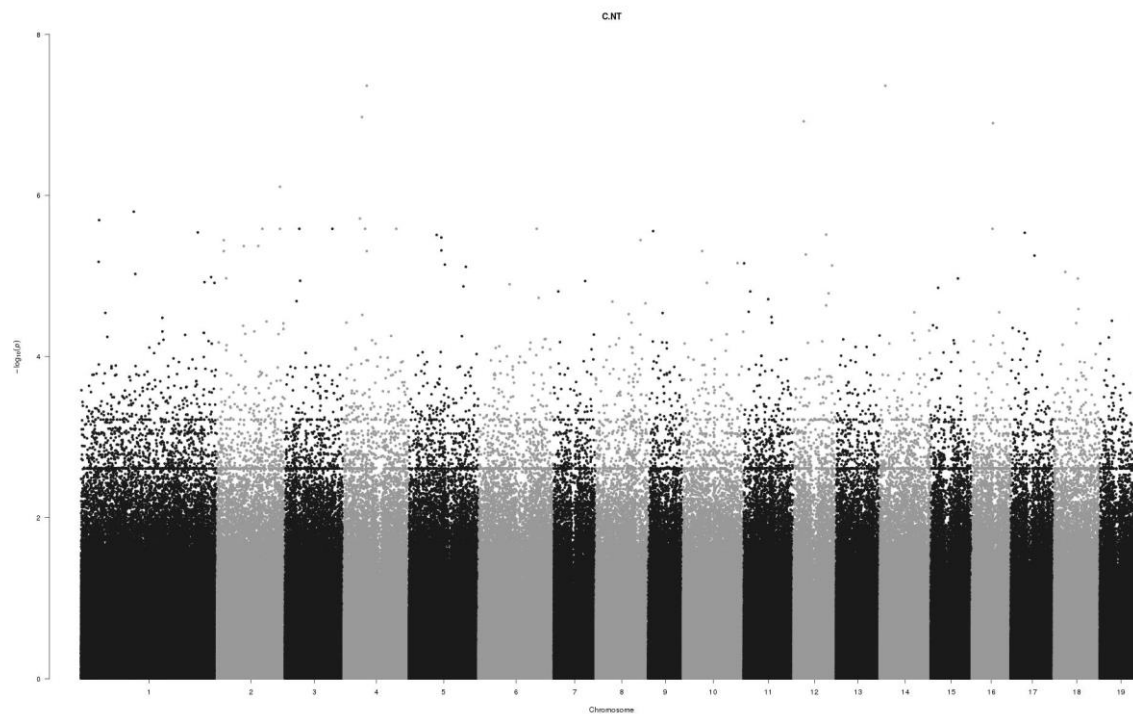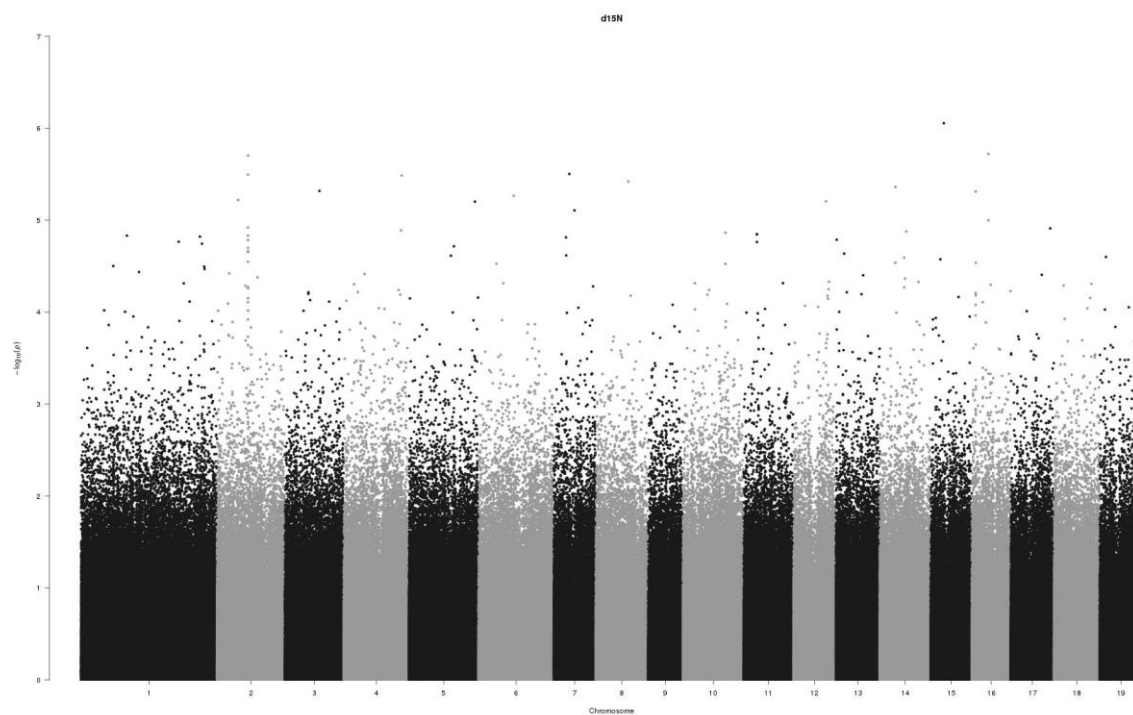

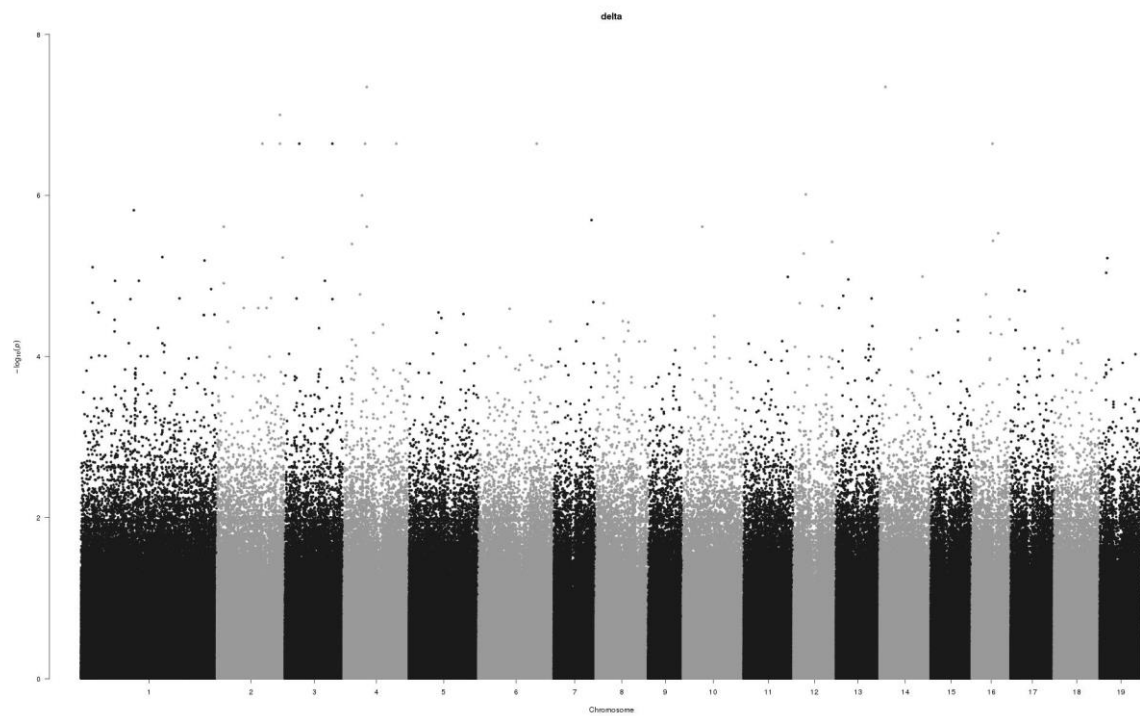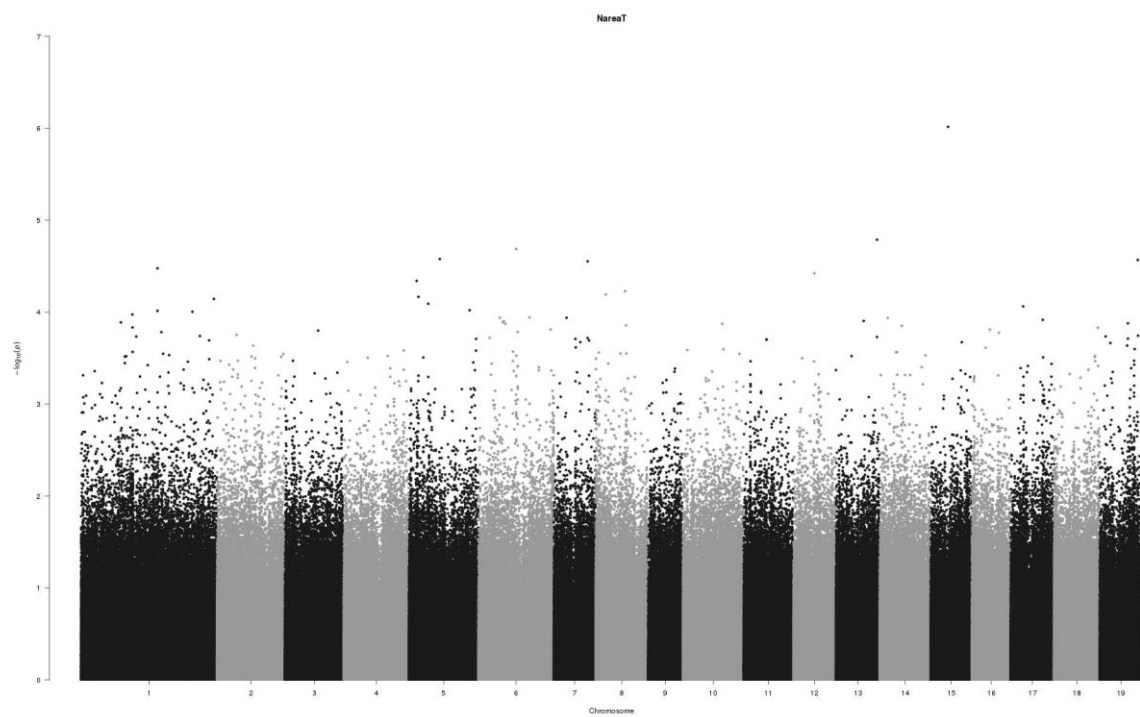

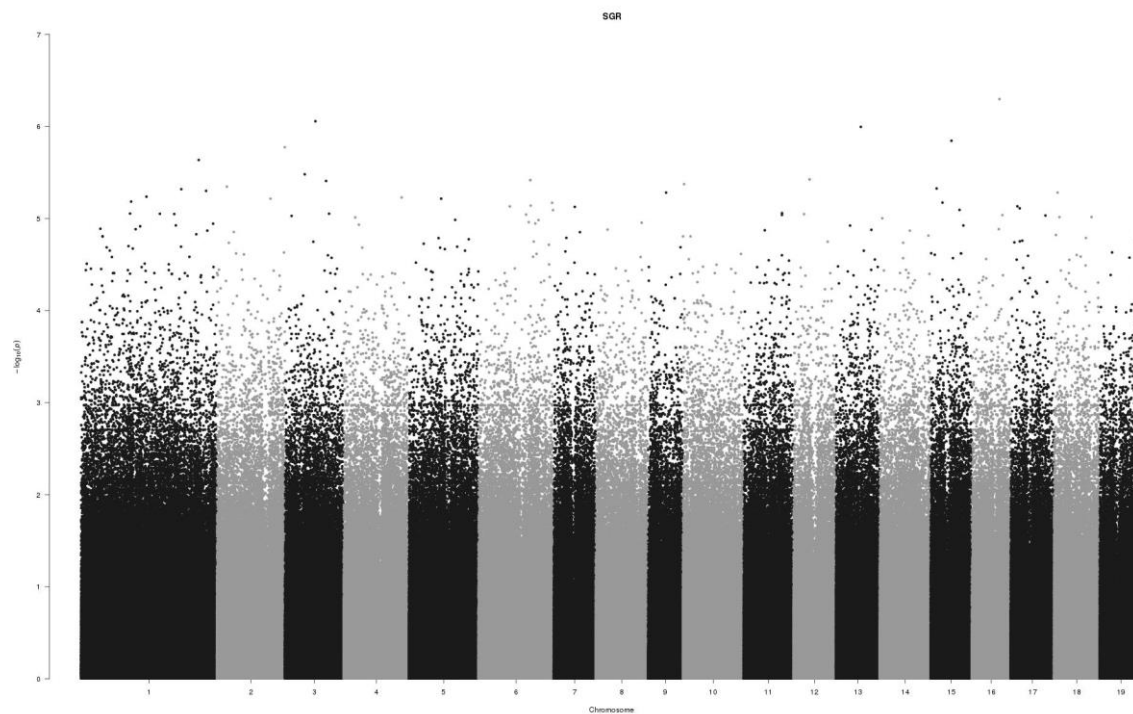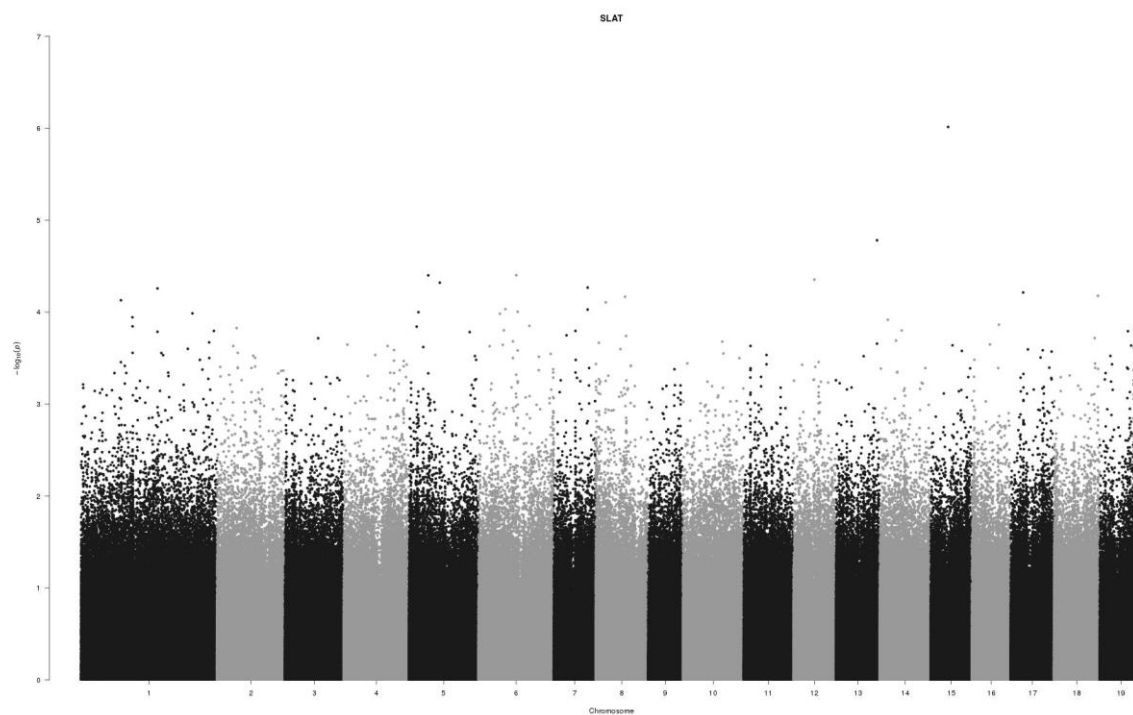

X\_CT

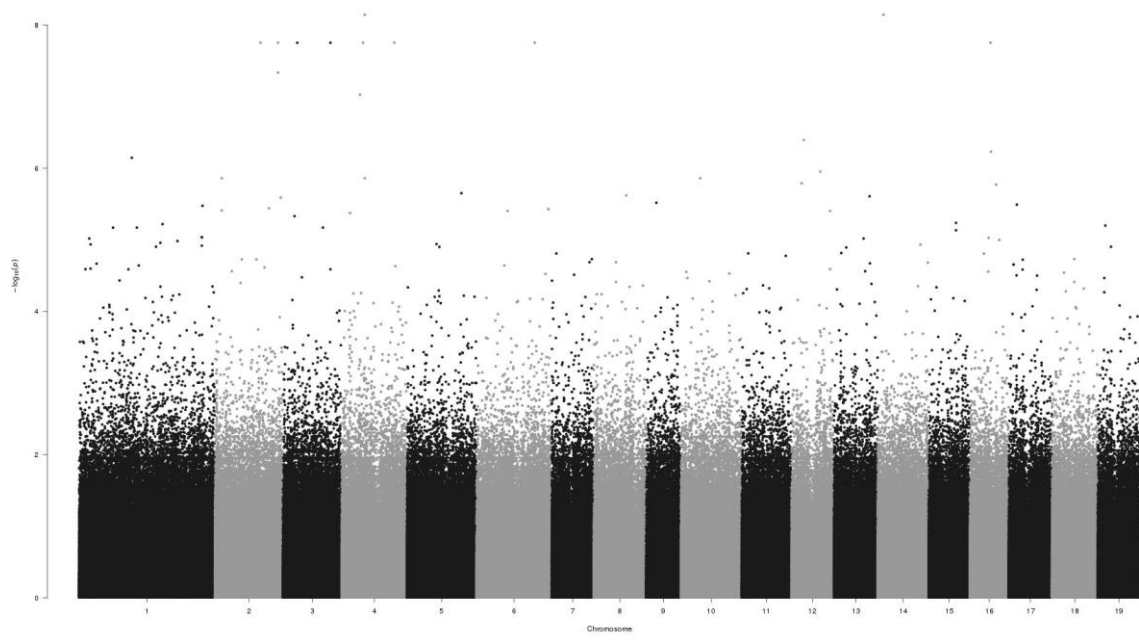

X\_NT

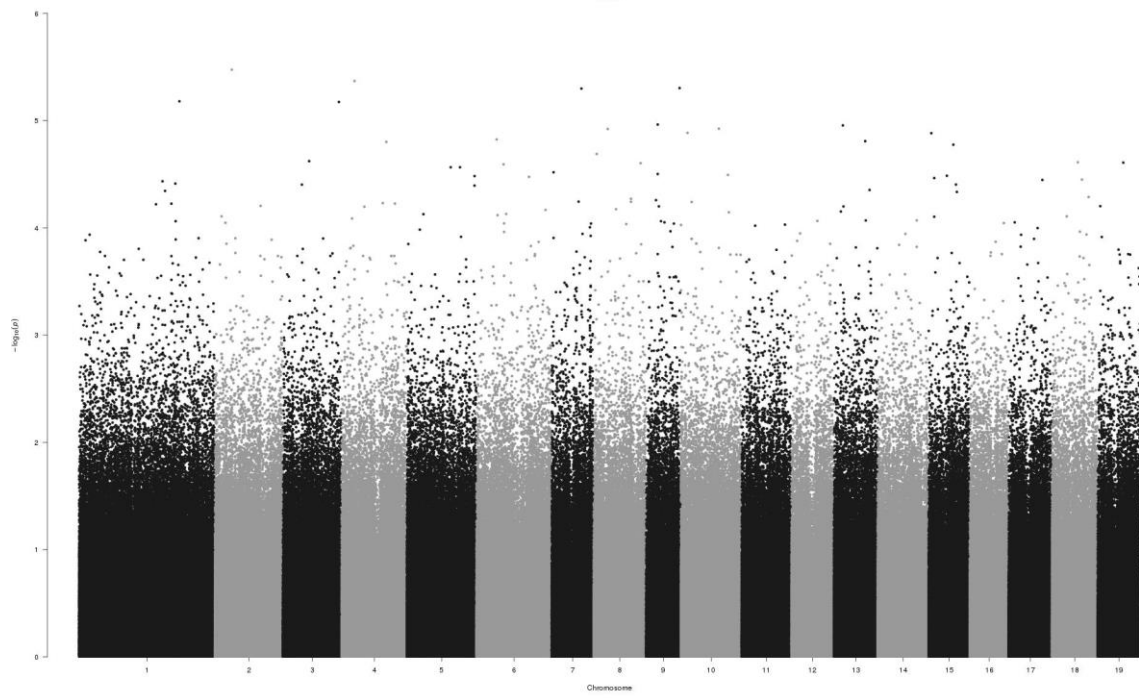

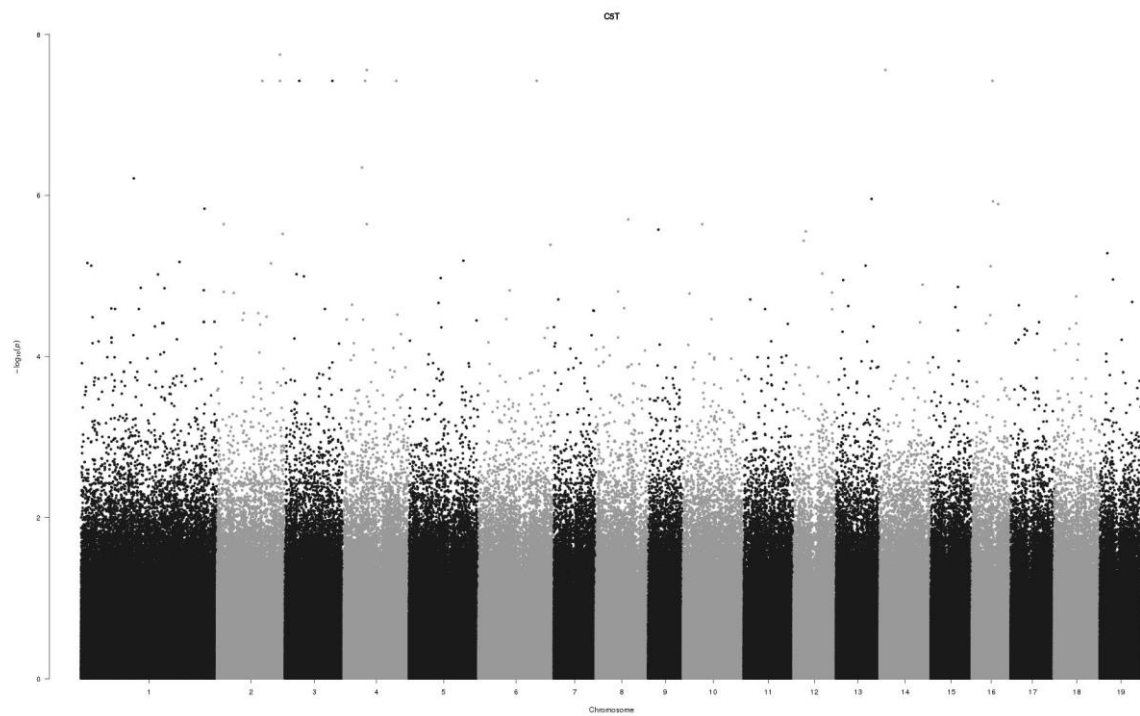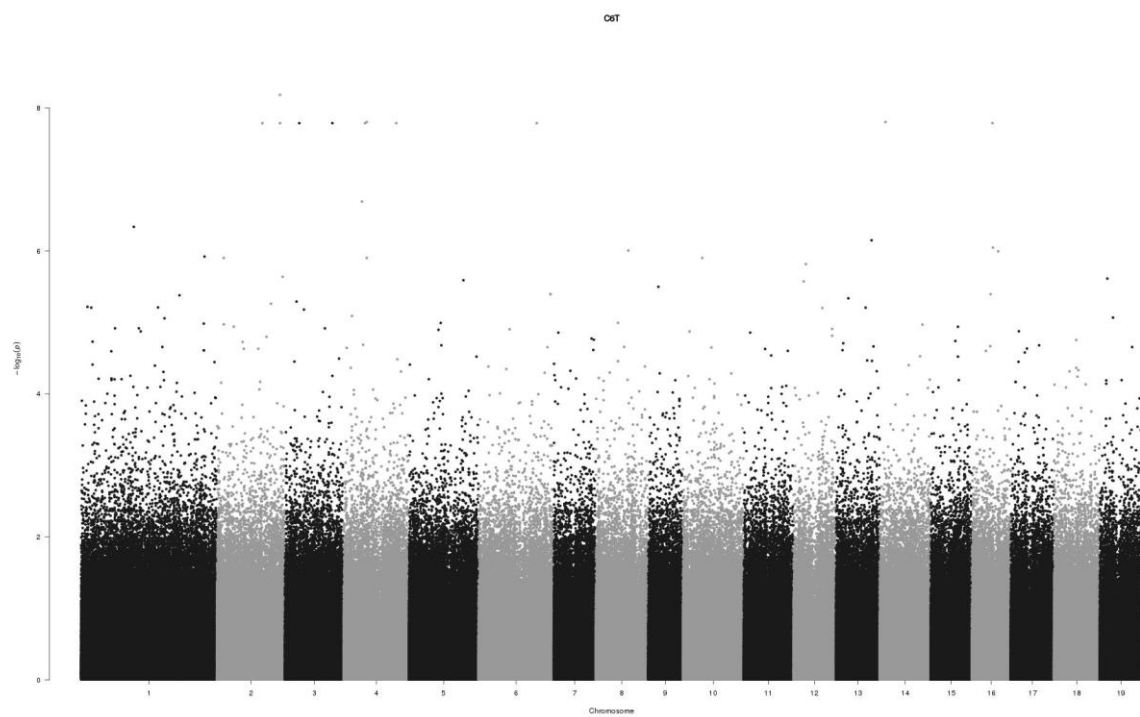

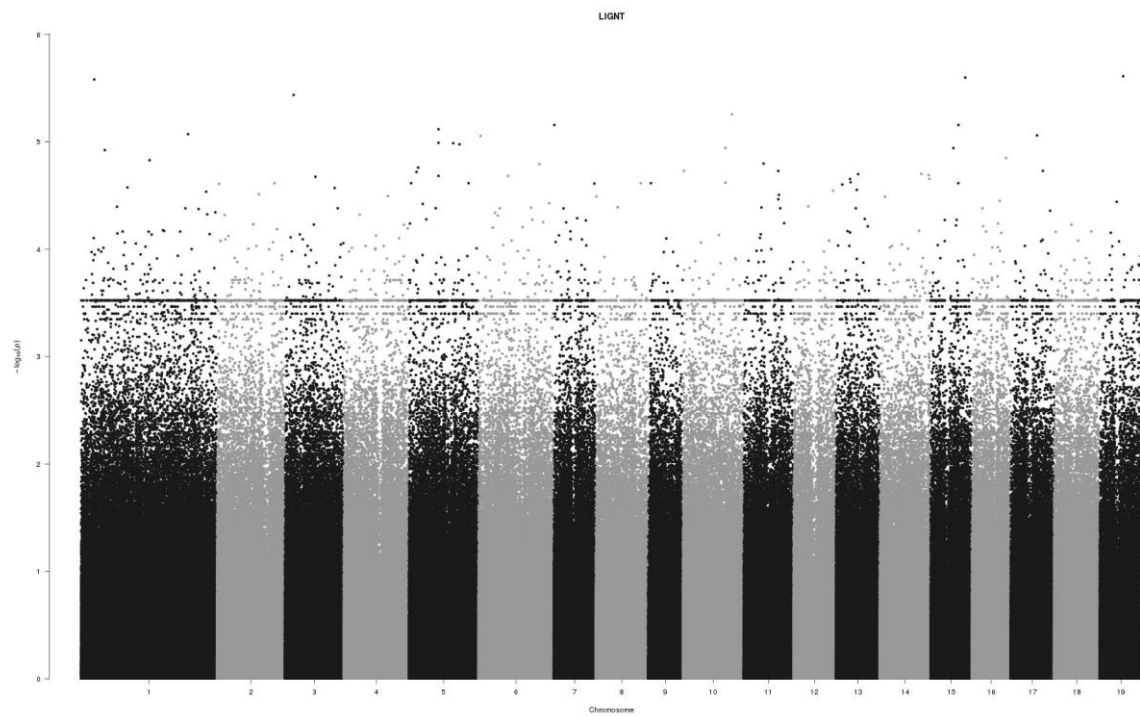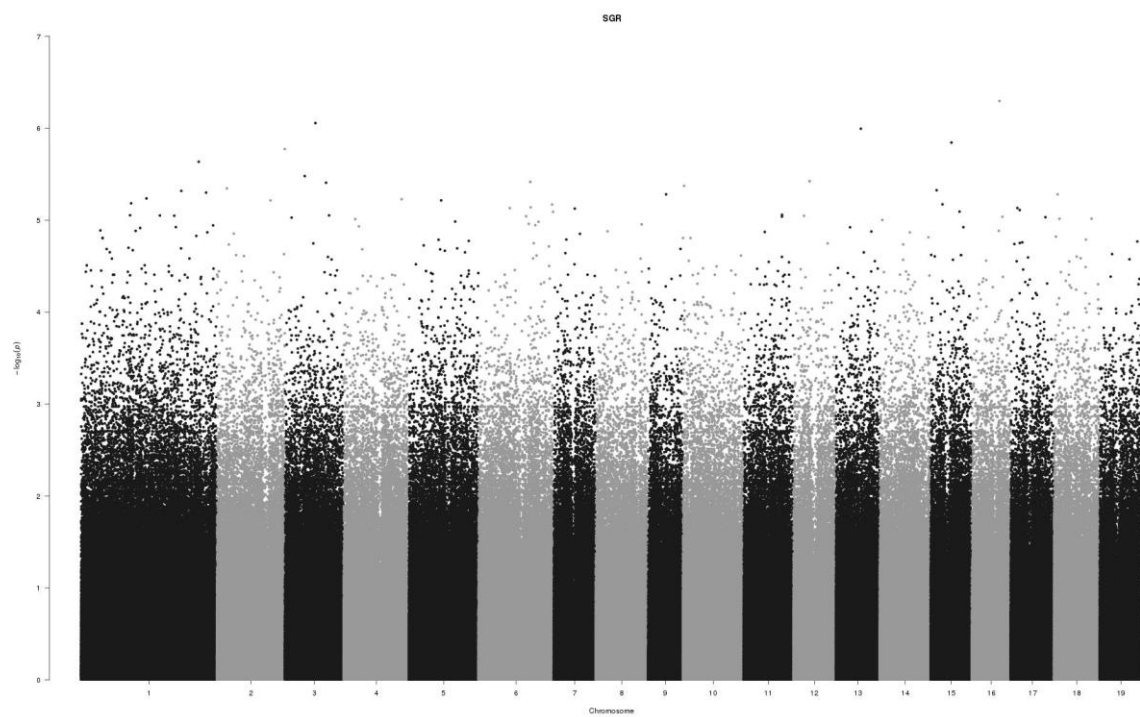

Supplement: Supplementary file 4 — Additional file 4: Figure S2. Manhattan plots for assessed traits. [file 12864_2019_6160_MOESM4_ESM.pdf]

**Figure S3.** Manhattan plots for sliding window analysis tests.

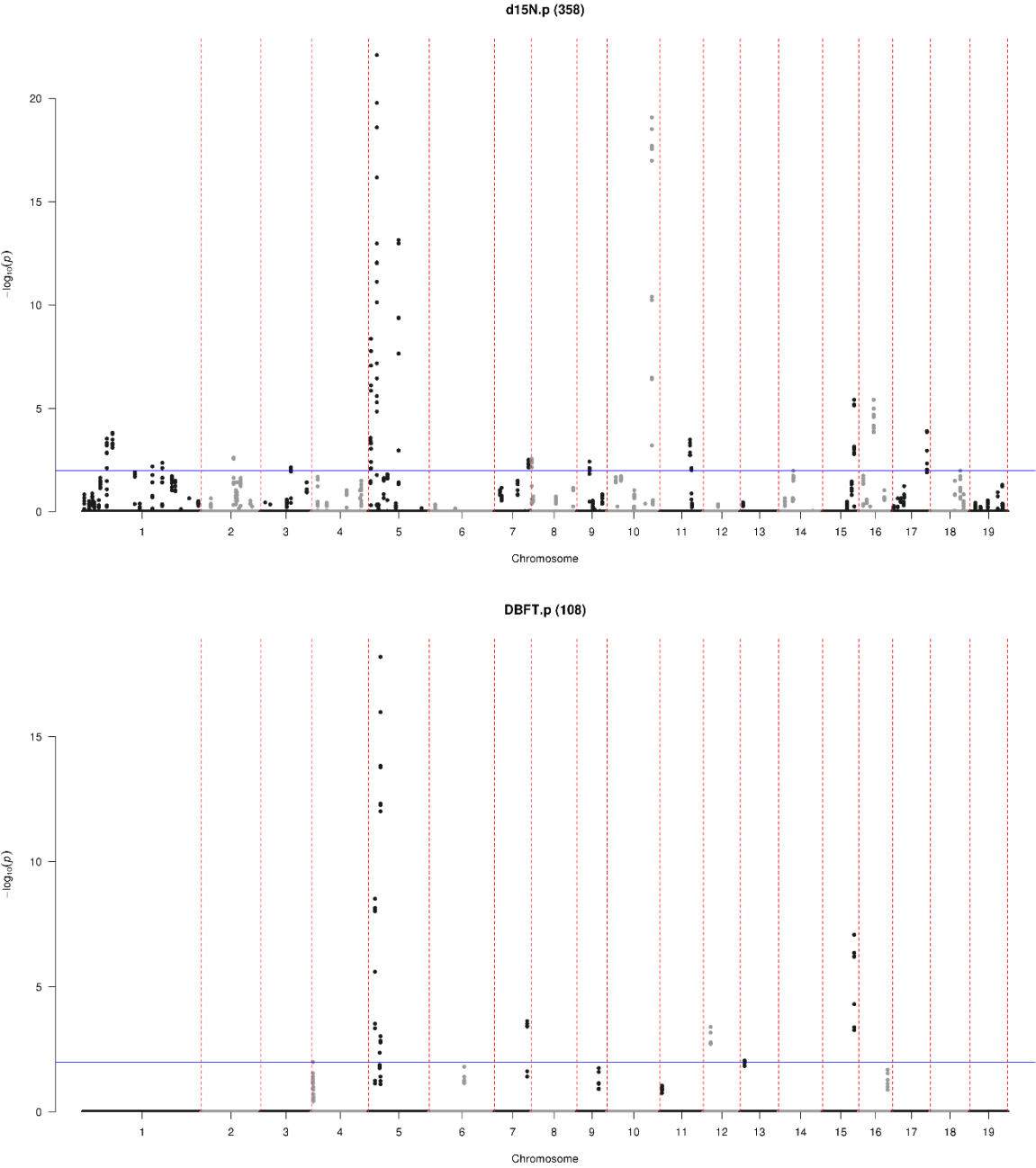

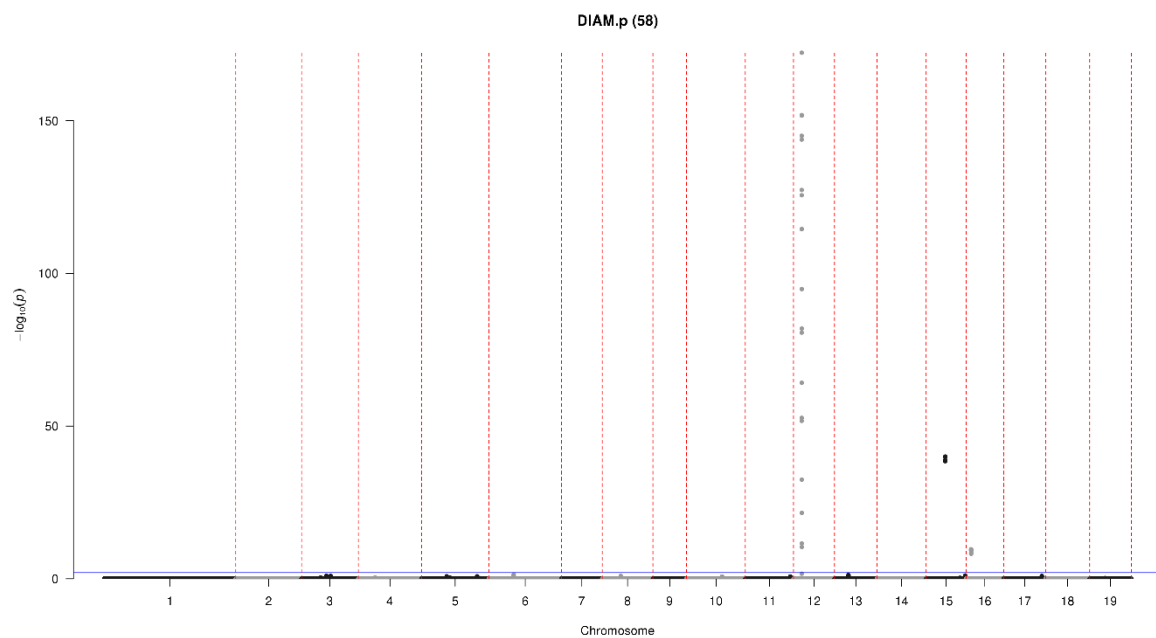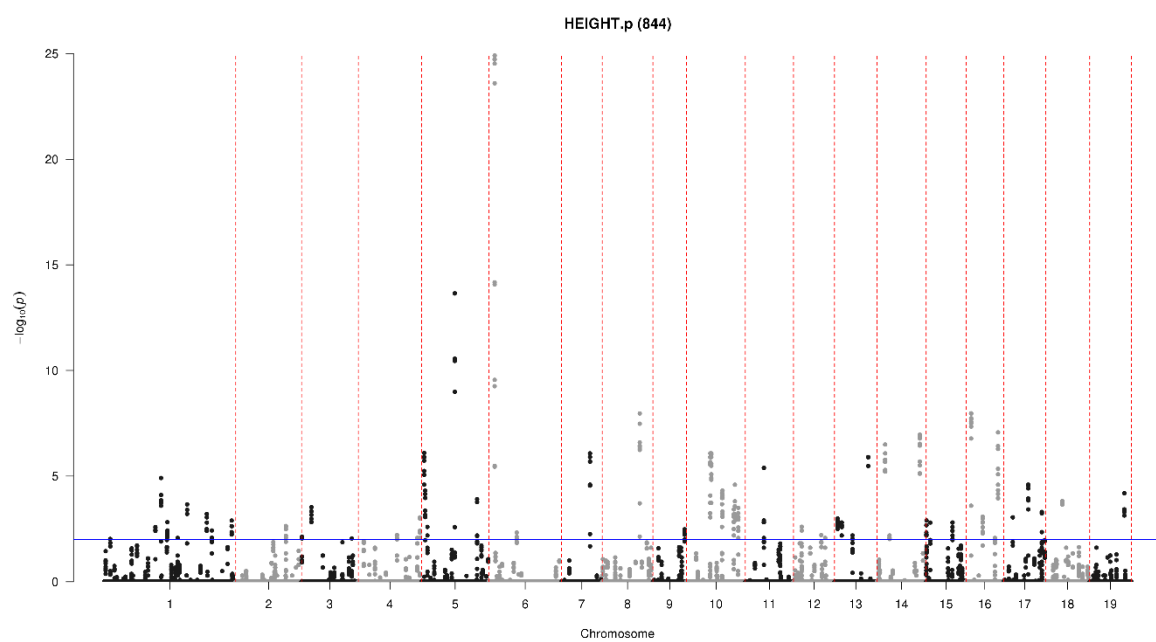

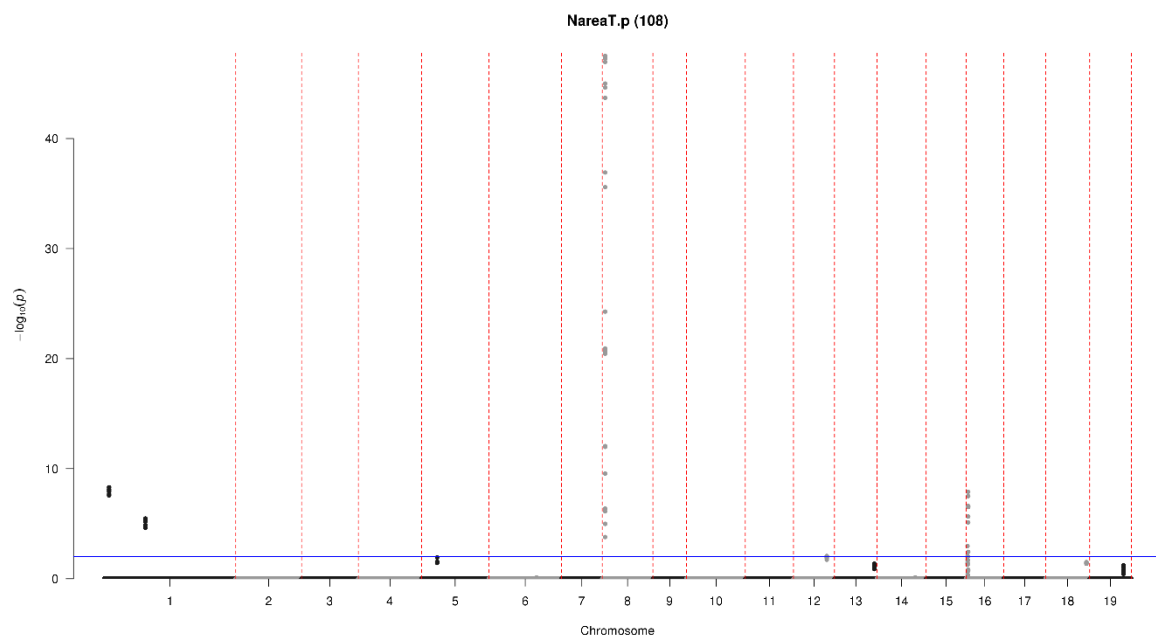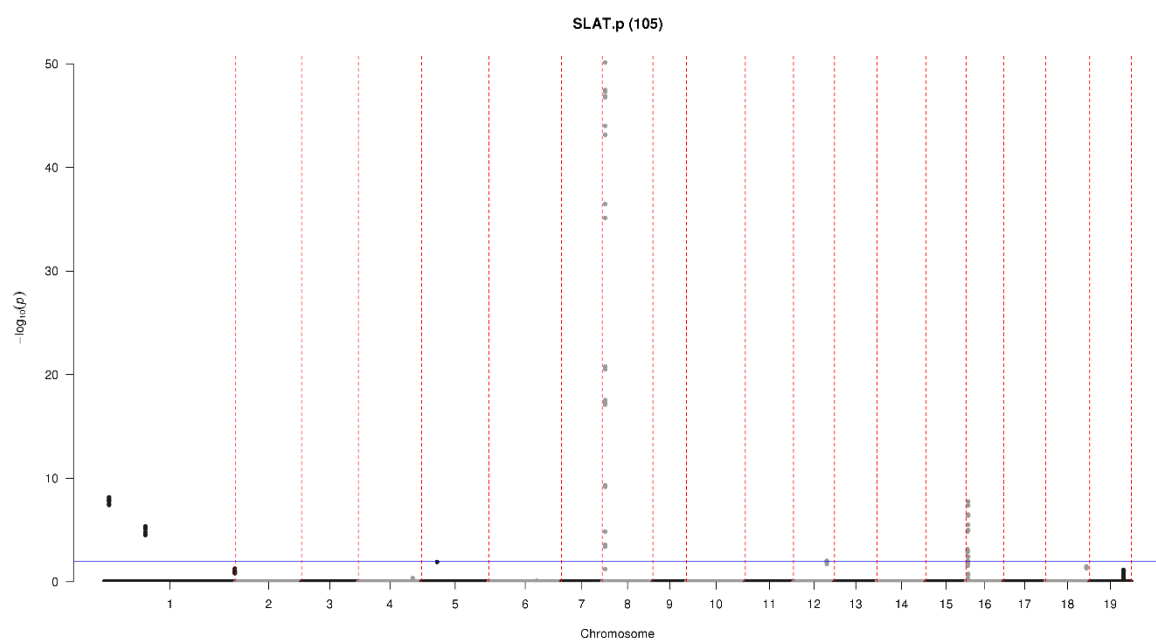

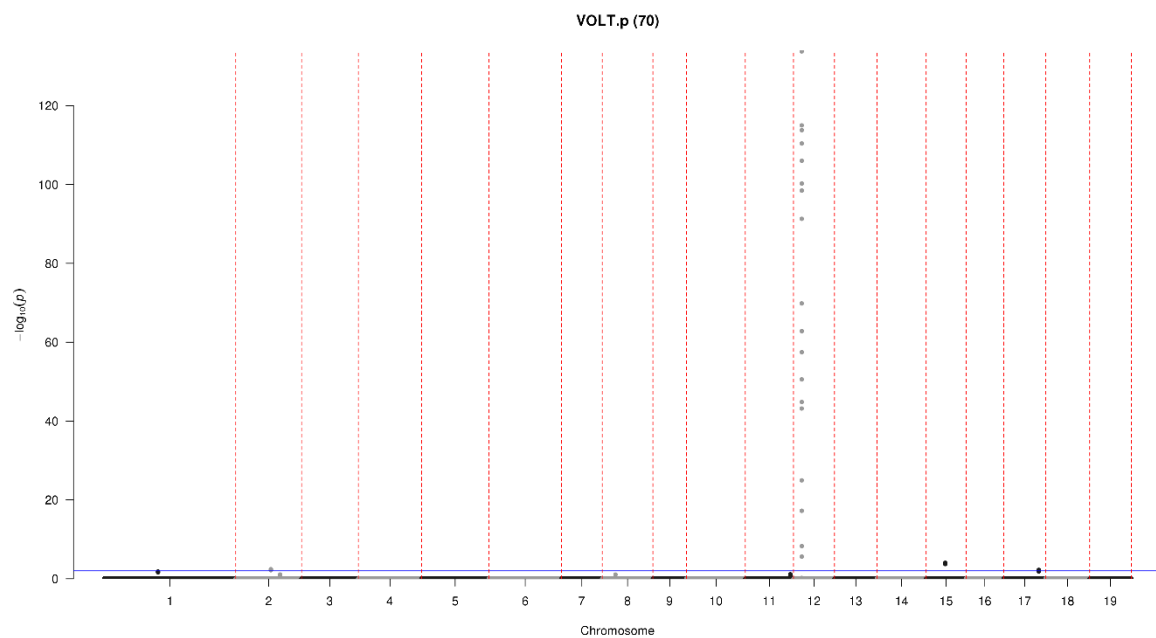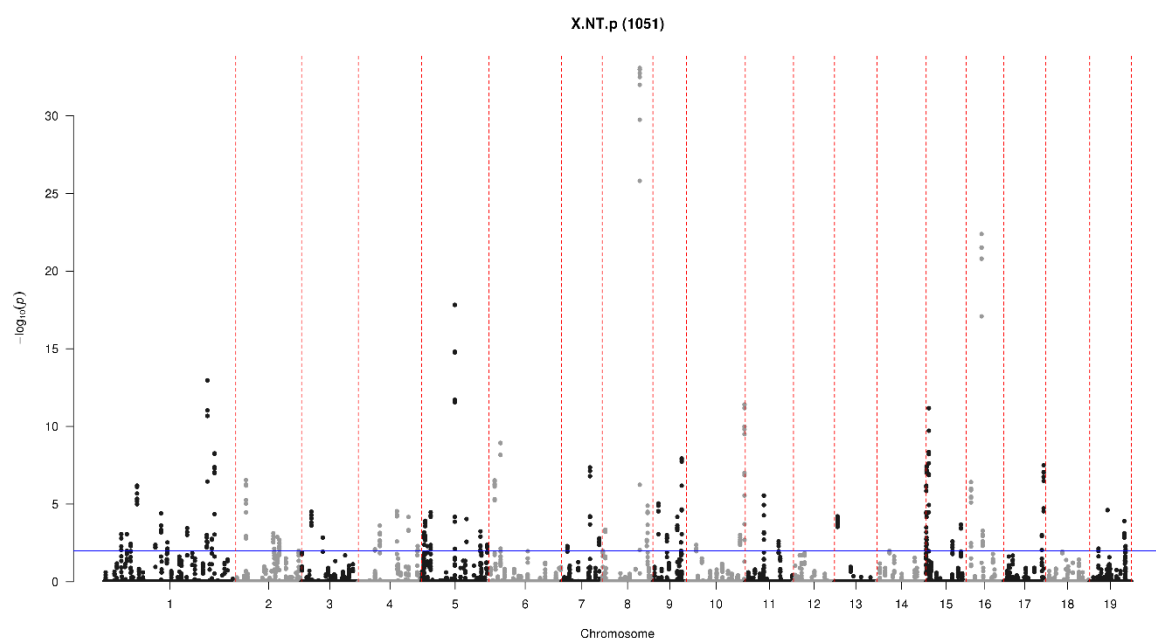

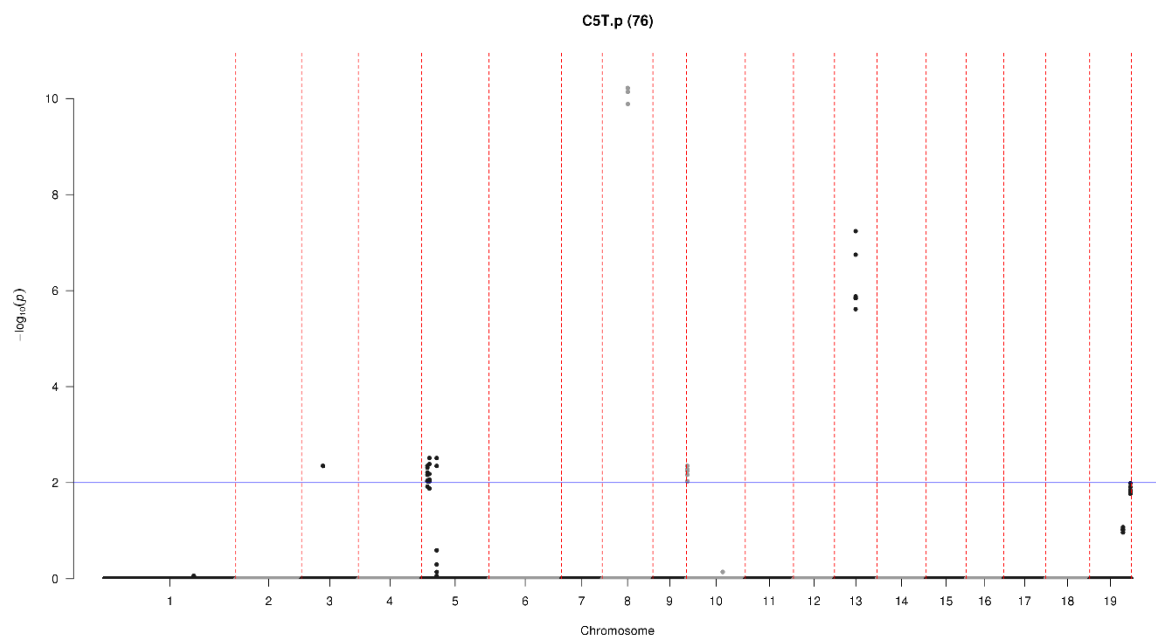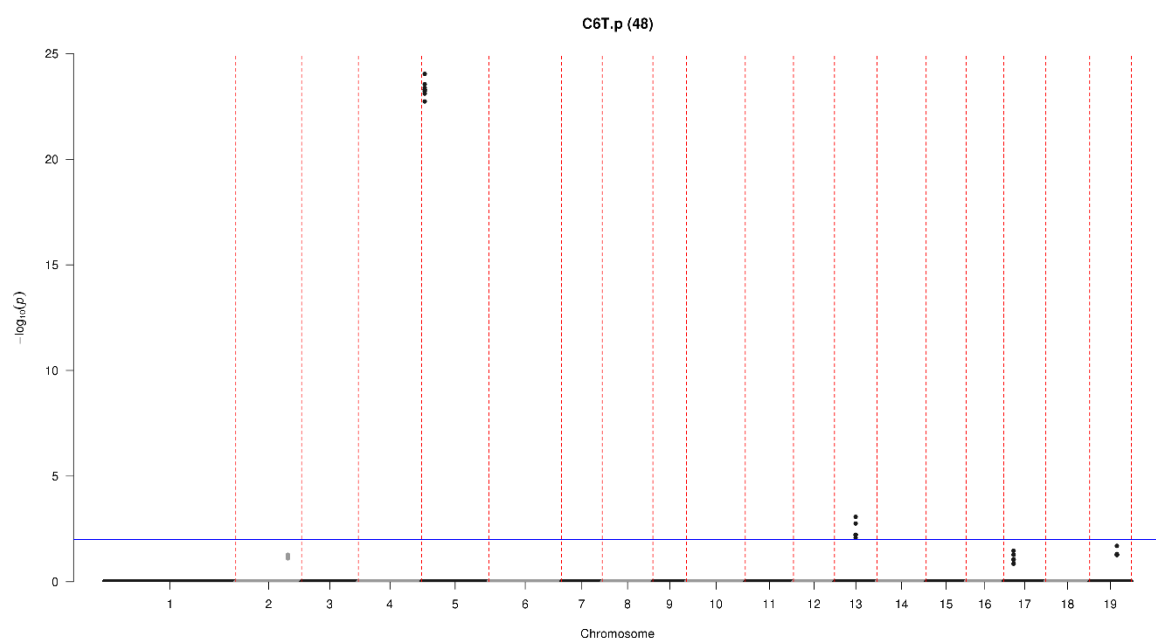

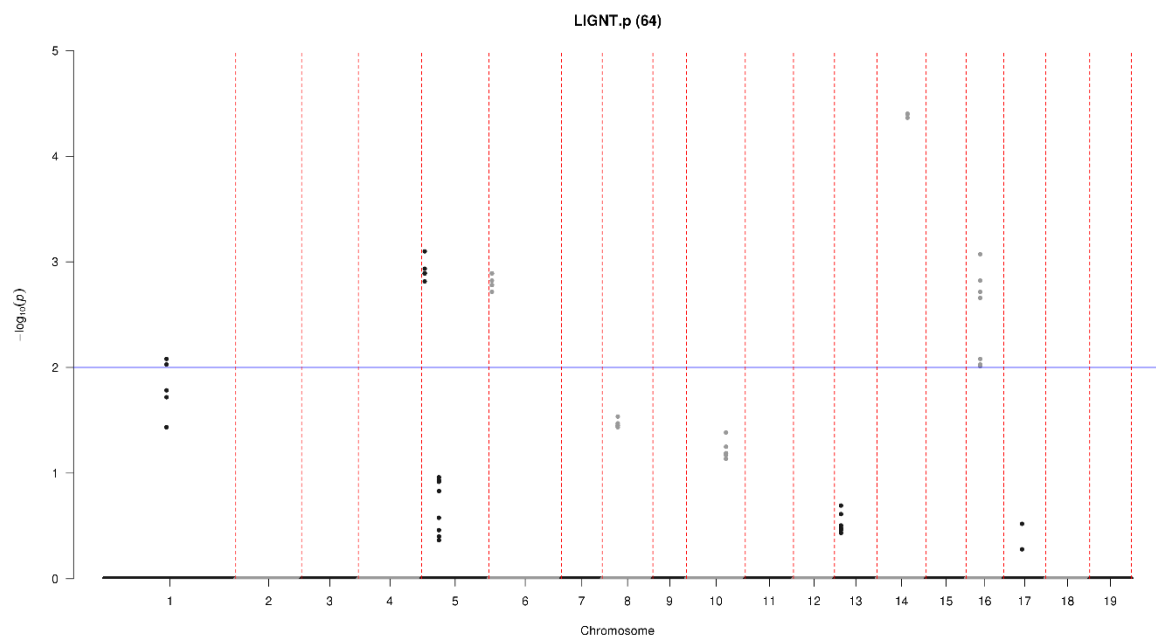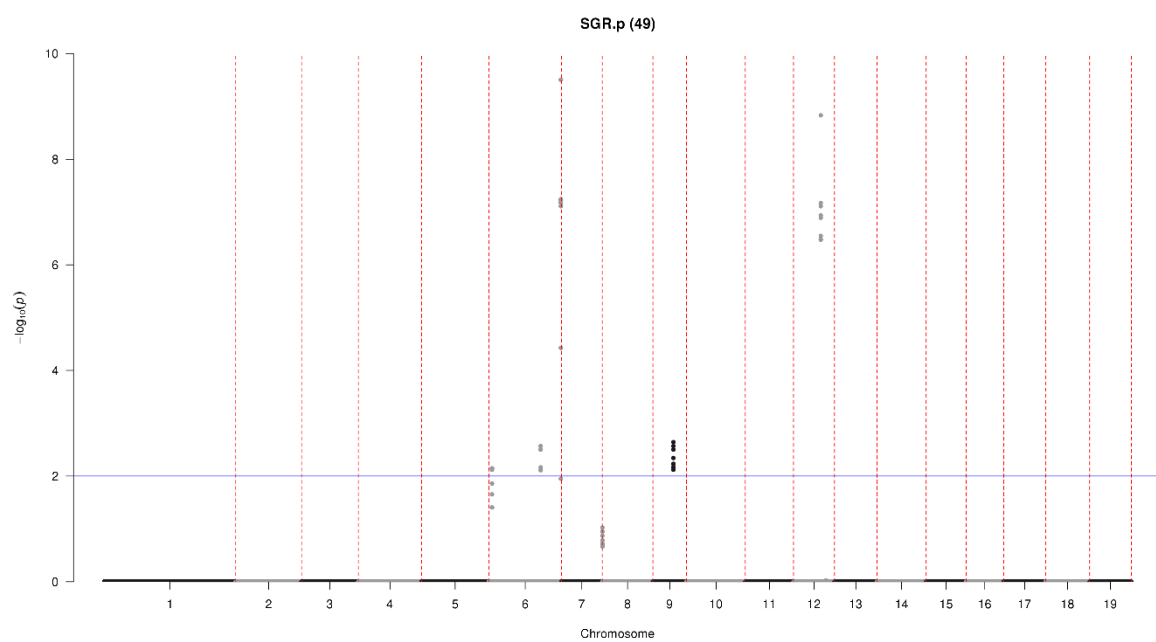

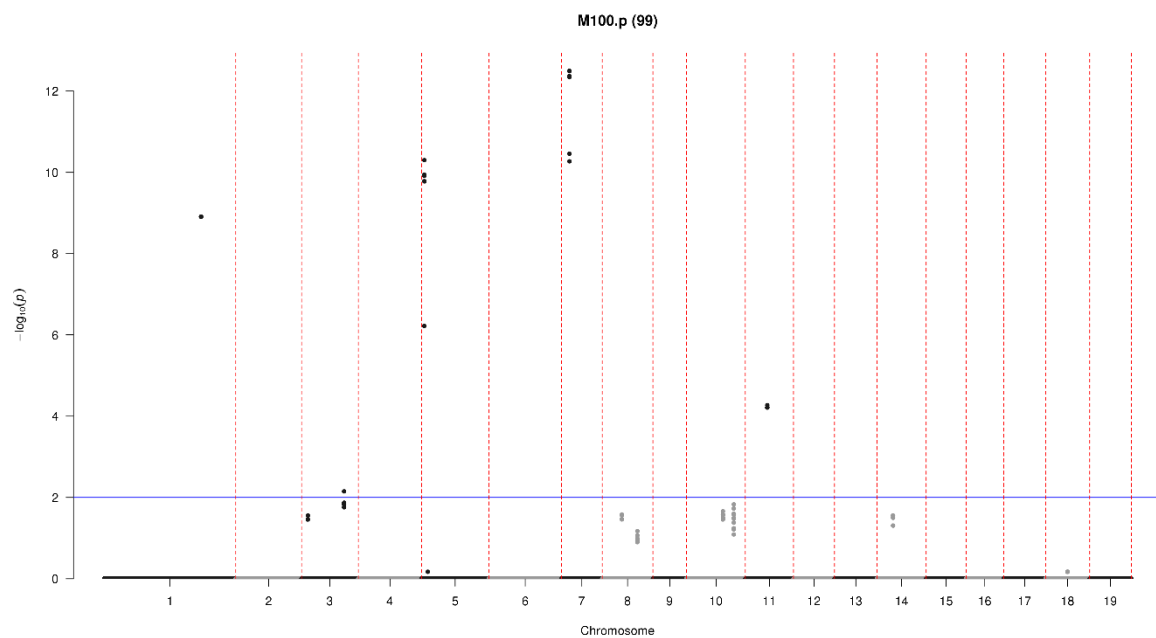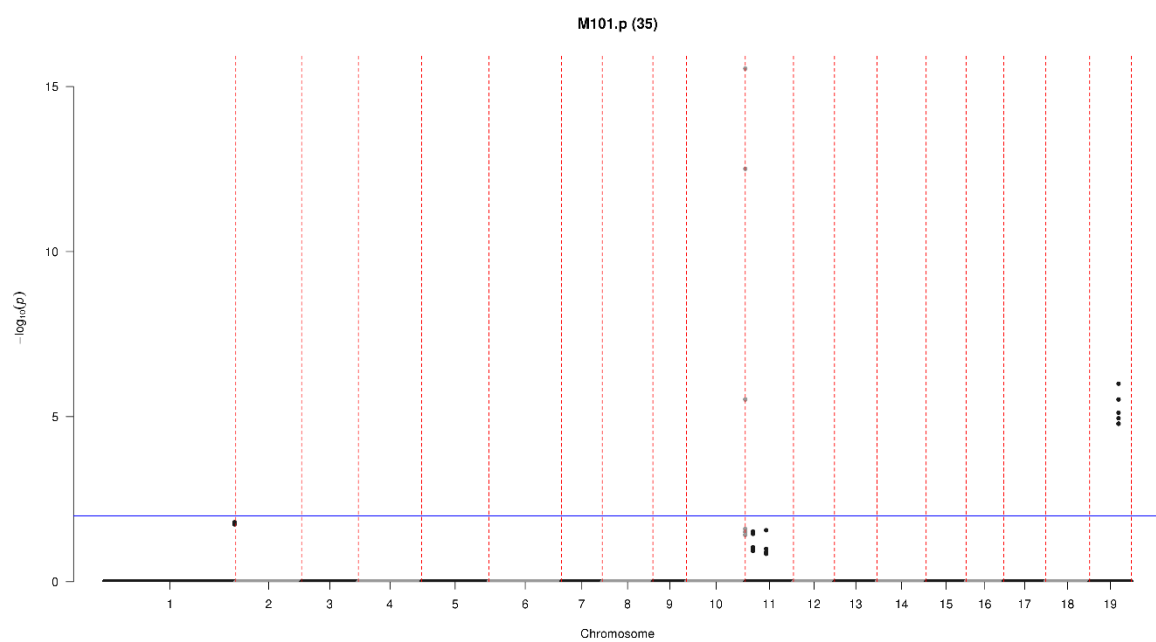

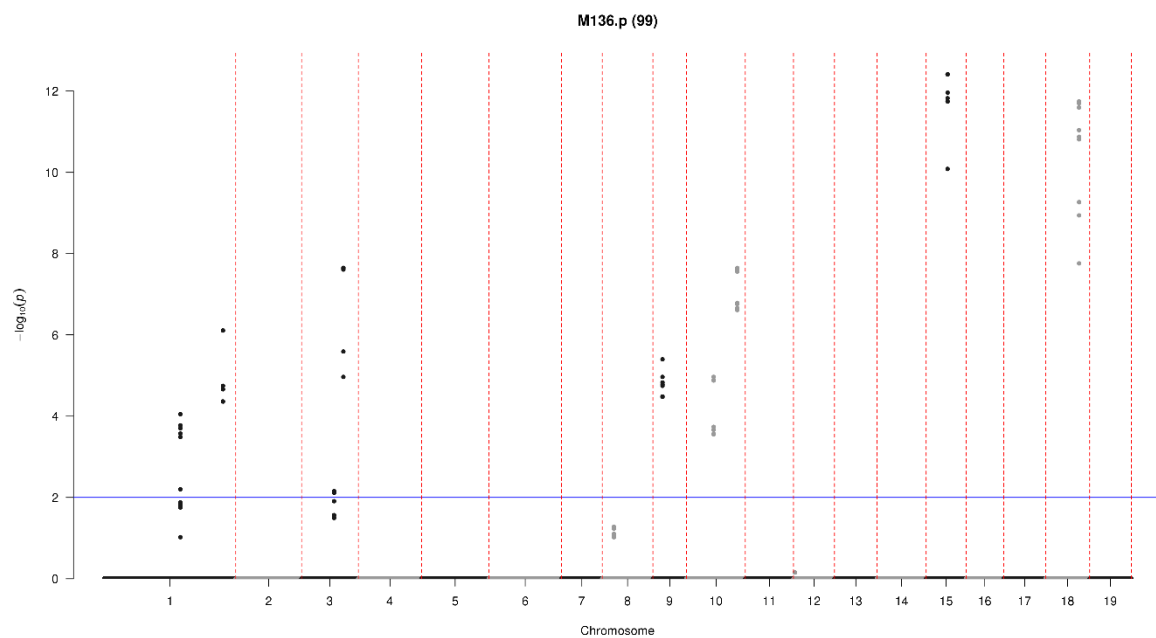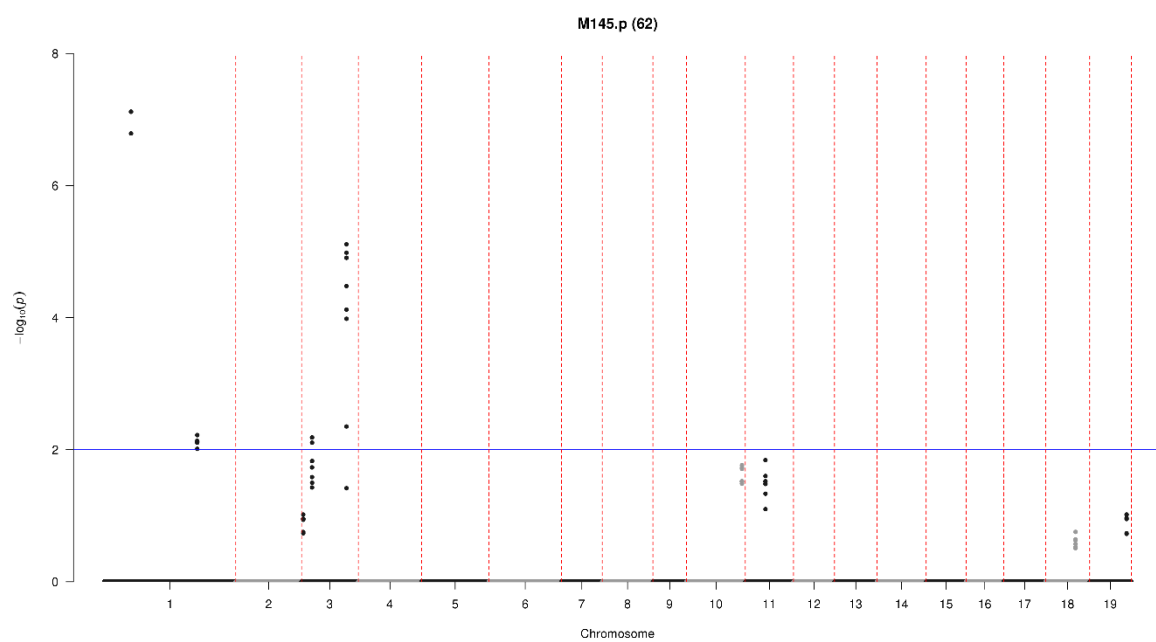

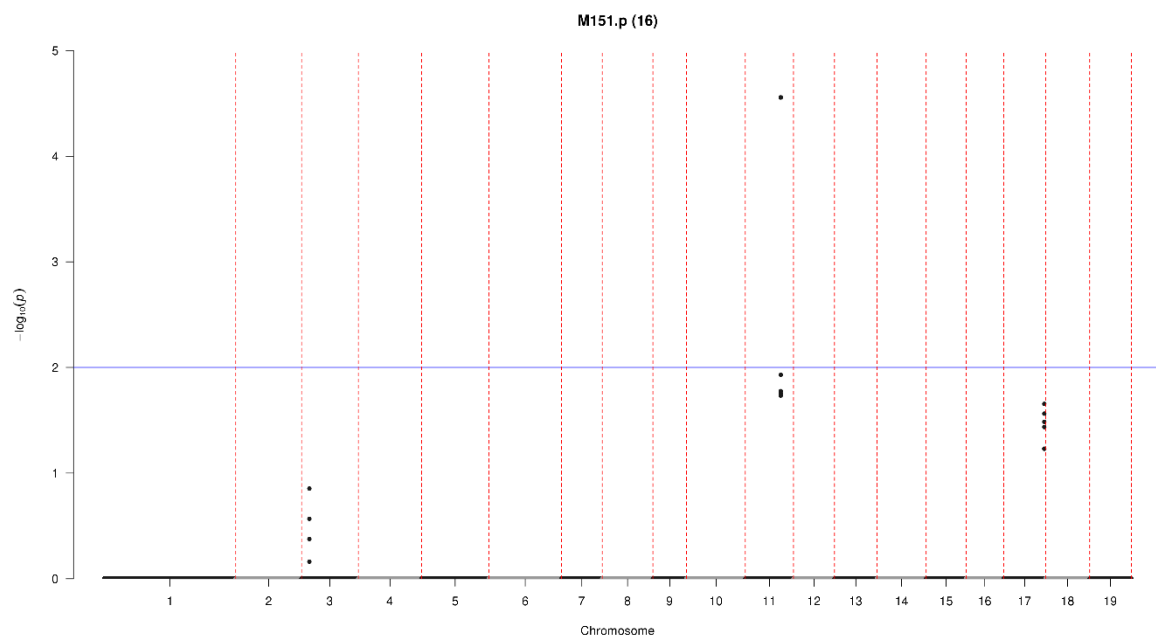

Supplement: Supplementary file 5 — Additional file 5: Figure S3. Manhattan plots for sliding window analysis tests. [file 12864_2019_6160_MOESM5_ESM.pdf]
